# Pathological impact of *SMN2* mis-splicing in adult SMA mice

**DOI:** 10.1002/emmm.201302567

**Published:** 2013-09-09

**Authors:** Kentaro Sahashi, Karen K Y Ling, Yimin Hua, John Erby Wilkinson, Tomoki Nomakuchi, Frank Rigo, Gene Hung, David Xu, Ya-Ping Jiang, Richard Z Lin, Chien-Ping Ko, C Frank Bennett, Adrian R Krainer

**Affiliations:** 1 Cold Spring Harbor LaboratoryCold Spring Harbor, NY, USA; 2 Section of Neurobiology, Department of Biological Sciences, University of Southern CaliforniaLos Angeles, CA, USA; 3 Department of Pathology, University of MichiganAnn Arbor, MI, USA; 4 Isis PharmaceuticalsCarlsbad, CA, USA; 5 Columbia University College of Physicians and SurgeonsNew York, NY, USA; 6 Department of Physiology and Biophysics and Institute of Molecular Cardiology, Stony Brook UniversityStony Brook, NY, USA

**Keywords:** adult-onset SMA, pathology, SMN2, spinal muscular atrophy, splicing

## Abstract

Loss-of-function mutations in *SMN1* cause spinal muscular atrophy (SMA), a leading genetic cause of infant mortality. The related *SMN2* gene expresses suboptimal levels of functional SMN protein, due to a splicing defect. Many SMA patients reach adulthood, and there is also adult-onset (type IV) SMA. There is currently no animal model for adult-onset SMA, and the tissue-specific pathogenesis of post-developmental SMN deficiency remains elusive. Here, we use an antisense oligonucleotide (ASO) to exacerbate *SMN2* mis-splicing. Intracerebroventricular ASO injection in adult *SMN2*-transgenic mice phenocopies key aspects of adult-onset SMA, including delayed-onset motor dysfunction and relevant histopathological features. *SMN2* mis-splicing increases during late-stage disease, likely accelerating disease progression. Systemic ASO injection in adult mice causes peripheral *SMN2* mis-splicing and affects prognosis, eliciting marked liver and heart pathologies, with decreased IGF1 levels. ASO dose–response and time-course studies suggest that only moderate SMN levels are required in the adult central nervous system, and treatment with a splicing-correcting ASO shows a broad therapeutic time window. We describe distinctive pathological features of adult-onset and early-onset SMA.

## INTRODUCTION

SMA is characterized by skeletal-muscle weakness and wasting, due to α-motor neuron degeneration, and no effective therapy is currently available. Loss-of-function mutations or deletion of *SMN1* and the resulting deficiency in the encoded SMN protein, which mediates snRNP assembly, cause SMA, although how this specifically affects α-motor neurons remains unclear (Burghes & Beattie, 2009). A closely related gene, *SMN2*, is present only in humans. Although *SMN2* exon 7 is predominantly skipped by alternative splicing, which results in a truncated defective protein, called SMNΔ7, *SMN2* acts as a disease modifier and reduces SMA severity as its copy number increases (McAndrew et al, 1997). Based on the age of onset and clinical severity, SMA is subdivided into types I, II, III and IV, with type I being the most severe form. Types I–III affect infants and children usually under the age of 3, whereas type IV shows adult onset (Lunn & Wang, 2008).

Several SMA models have been generated to reproduce SMA with various severities. Knockout of the murine *Smn* gene results in embryonic lethality (Schrank et al, 1997). Introduction of a human *SMN2* transgene rescues this phenotype, such that *Smn*^−/−^ *SMN2* mice have SMA-like phenotypes whose severity inversely correlates with the *SMN2* copy number (Hsieh-Li et al, 2000; Monani et al, 2000). Severe-SMA mice harbouring two *SMN2* copies, or with an extra SMNΔ7 cDNA transgene (SMA Δ7 mouse model), develop early and rapidly progressive pathology, dying within 1–2 weeks postnatally (Hsieh-Li et al, 2000; Le et al, 2005; Monani et al, 2000; Riessland et al, 2010). In contrast, SMA mice harbouring four *SMN2* copies survive normally and do not develop paralysis, but have an abnormal, short and thick tail and develop tail and ear necrosis, beginning around 3 weeks and 3 months postnatally, respectively (Hsieh-Li et al, 2000). These models provide distinct advantages, including the testing of therapeutic strategies based on targeting the human *SMN2* transgene by means of splicing correction or upregulation (Park et al, 2010a).

RNA splicing requires pre-mRNA *cis*-acting elements recognized by *trans*-acting factors, such as spliceosome components and auxiliary RNA-binding proteins (Cartegni et al, 2002). Antisense oligonucleotides (ASOs) can be designed to target *cis*-element(s) on a given pre-mRNA, so as to preclude binding of *trans*-acting factors and thereby modulate splicing patterns. These properties enable the development of RNA-targeted therapeutics to correct disease-associated splicing defects or restore the translational reading frame (Bennett & Swayze, 2010). We previously reported that a 2′-*O*-(2-methoxyethyl) (MOE) therapeutic ASO (ASO-10-27 or ISIS-SMN_Rx_) that promotes exon 7 inclusion in *SMN2*, rescues the phenotypes of several SMA mouse strains (Hua et al, 2010, 2011; Passini et al, 2011; Sahashi et al, 2012). Recently, a phase Ib/IIa clinical study of ASO-10-27 has been initiated in children with intermediate SMA.

We have also employed ASO technology to phenocopy SMA in transgenic mice, utilizing ASOs that exacerbate the *SMN2* splicing defect and persistently promote pathogenesis. Intracerebroventricular (ICV) administration of an exon-7-complementary MOE ASO (ASO-20-37) that promotes *SMN2* exon 7 skipping in neonatal four-copy *SMN2*-transgenic mice successfully phenocopies intermediate SMA, including ∼1-month lifespan and progressive motor dysfunction, with α-motor neuron loss and abnormal neuromuscular junctions (NMJs) (Sahashi et al, 2012). These phenotypes are shared by other intermediate SMA models with point mutations (*Smn*^2B/−^ mice) or exon deletions (*Smn*^F7/Δ7^, NSE-Cre mice) in murine *Smn*, although these strains lack an *SMN2* transgene, which is being actively pursued as a therapeutic target in human SMA (Park et al, 2010a).

Available SMA mouse strains, including those with inducible expression of SMN, are extremely useful for studying the temporal and spatial requirements for SMN (Gavrilina et al, 2008; Le et al, 2011; Lutz et al, 2011; Park et al, 2010b), although the physiological roles of SMN and pathological roles of SMN deficiency after the developmental stages, remain unclear. A recent report showed that removal of ectopic SMN induction after postnatal Day 28 in an SMA Δ7 mouse background resulted in some of the mice surviving for >8 months (Le et al, 2011). However, the tissue-specific effects of adult-onset SMN deficiency have not been addressed. Many SMA patients reach adulthood, and there is an adult-onset form of the disease, namely type IV SMA, characterized by progressive paralysis and decline in daily-living activities. Therefore, addressing the effect of SMN levels and the phenotypic effects of SMN deficiency/restoration in adult mice should contribute to the understanding of SMA pathogenesis and to the development of targeted therapies. Animal models of adult-onset SMA would be extremely valuable for such studies.

Here we extended our antisense exon-skipping approach to adult mice with four copies of an *SMN2* transgene. We found that ICV-administered ASO phenocopies adult-onset SMA. The extent of *SMN2* mis-splicing in the central nervous system (CNS) determined the severity of the SMA-like motor symptoms. *SMN2* mis-splicing was exacerbated during late-stage disease, which should accelerate the decline. In addition, systemically administered exon-skipping ASO also affected survival, resulting in striking liver and heart lesions, and the combination of central and peripheral administration exacerbated the pathology. We demonstrated effective rescue with therapeutic ASO-10-27, suggesting that there is a broad temporal therapeutic window for treatment of adult-onset SMA. The ability to persistently modulate splicing of a target gene using ASOs provides a powerful method to model and characterize diseases in animals.

## RESULTS

### Inhibition of *SMN2* splicing in mouse tissues

To address the post-developmental roles of SMN deficiency in SMA pathogenesis, and to develop a mouse model for adult-onset SMA, we attempted to increase skipping of exon 7 in *SMN2* transgene pre-mRNA in transgenic mice with four *SMN2* copies (*Smn*^−/−^ *SMN2*^+/+^) using ASOs. This mouse model has normal motor function and >2-year lifespan, but develops postnatal, progressive necrosis of the tail and ear pinna (Hsieh-Li et al, 2000). We could then assess the tissue-specific effects of *SMN2* mis-splicing in this adult-mouse context.

**Figure 1:**
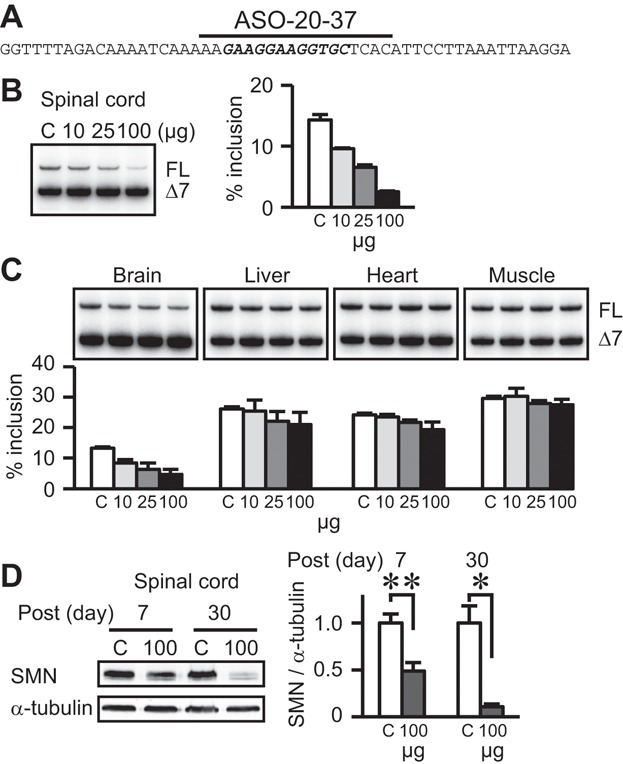
ASO-induced inhibition of *SMN2* splicing and SMN expression FL and Δ7: full-length and exon-7-skipped transcripts, respectively. % Inclusion: the percentage of FL in total transcripts. C: ICV injection of 100 µg control ASO. We analysed data using two-tailed *t* tests. **A.** *SMN2* exon 7 sequence and schematic representation of ASO-20-37. The splicing-enhancer region is in bold–italic font, and the bar depicts ASO-20-37 above its complementary sequence.**B,C.** Dose–response study of ASO-20-37 effects (*n* = 3). RT-PCR shows inhibition of exon 7 inclusion in PS7 spinal cord (B) and other tissues (C).**D.** Western analysis shows reduction in SMN expression at PS7 and especially at PS30. For full-length SMN, the upper band in each doublet was quantitated as described (*n* = 3. *p = 0.0029; **p = 0.0005; Sahashi et al, 2012).

Based on a screen of overlapping ASOs tiled along *SMN2* exon 7 and the flanking introns (Hua et al, 2007, 2008), we previously characterized ASO-20-37, which targets splicing enhancer elements recognized by TRA2-β1 and another presumptive activator in exon 7 (Fig 1A; Supporting Information Table S1; Hofmann et al, 2000; Hua et al, 2007; Sahashi et al, 2012). This ASO substantially inhibits *SMN2* exon 7 inclusion in neonatal tissues of the transgenic mouse, and accurately phenocopies intermediate SMA when administered by ICV injection on postnatal Day 1 (P1). Moreover, the phenotype is ameliorated by ICV administration of therapeutic ASO-10-27 (Supporting Information Table S1), which targets a silencer in intron 7 and restores *SMN2* splicing (Sahashi et al, 2012). We routinely use synthetic ASOs with 2′-MOE modification of the ribose and phosphorothioate backbone, which do not trigger cleavage of the target RNA by RNase H or RNAi (Crooke, 2007).

Because SMA primarily affects α-motor neurons in the brainstem and spinal cord, and the blood–brain barrier (BBB) prevents the access of systemically administered MOE ASO to the CNS in adult mice (Crooke, 2007), we again used direct administration into the cerebral ventricles, such that the ASOs circulate in cerebrospinal fluid and distribute throughout the CNS. We used stereotaxic surgery to administer 10, 25 or 100 µg of ASO-20-37 as a single ICV injection, in 2-month-old adult *SMN2*-transgenic mice. We also injected 100 µg of a control ASO with six mismatches relative to ASO-10-27 (Supporting Information Table S1). We analysed the effect of the ASOs on *SMN2* splicing in the spinal cord 7 days post-surgery (PS7). Semi-quantitative radioactive RT-PCR showed that ASO-20-37 gave dose-dependent increases in exon 7 skipping at PS7, whereas the control ASO had no effect at either PS7 or PS14 (Fig 1B; Supporting Information Fig S1A). Immunoblot analysis showed a corresponding decrease in SMN protein expression in the spinal cord (Fig 1D). ICV-injected ASO-20-37 inhibited *SMN2* splicing in both the brain and spinal cord to a considerably higher extent than in peripheral tissues (Fig 1B and C), consistent with the ASO-uptake patterns observed in these tissues at PS7 by immunohistochemical (IHC) staining (Supporting Information Fig S2A).

**Figure 2:**
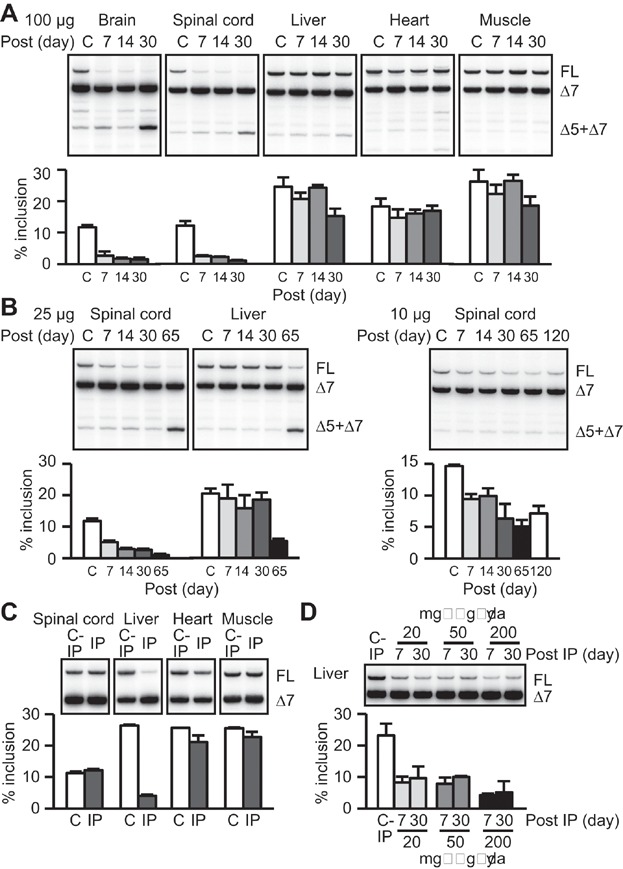
Time-course RT-PCR analysis of *SMN2* mis-splicing Δ5 + Δ7: mRNA lacking both exon 5 and exon 7. C: ICV injection of 100 µg in (A and B) or C-IP: IP injection of 200 mg/kg/day control ASO in (C and D). We analysed data using two-tailed *t* tests. ICV injection of 100 µg ASO-20-37. Further inhibition of exon 7 inclusion at PS30 except in heart, and increased simultaneous skipping of exons 5 and 7 in PS30 CNS were observed.ICV injection of 25 or 10 µg ASO-20-37. The skipping of exons 5 and 7 was observed in spinal cord and liver of PS65 mice treated with 25 µg ASO-20-37. For (A and B), *SMN2* splicing was analysed at PS7, 14, 30, 65 or 120 (*n* = 3). Bar graphs display the percentage of FL in FL plus Δ7.IP injection of 200 mg/kg/day ASO-20-37 inhibited *SMN2* splicing at PS7 in peripheral tissues, but not in spinal cord (*n* = 3).IP injection of 20 or 50 mg/kg/day ASO-20-37 had similar effects at PS7 or PS30 in liver, and both doses were less potent than 200 mg/kg/day (*n* = 3).

A time-course analysis showed that the splicing effect in the CNS of treatment with 100 µg ASO-20-37 was similar between PS7 and PS14 (Fig 2A), as seen also with therapeutic ASO-10-27, which has the same length and chemical modifications (Supporting Information Fig S1B). In the spinal cord, the effect of treatment with 25 µg ASO-20-37 on *SMN2* splicing plateaued after PS7. As expected, this dose had no significant effect on splicing in the liver, which we analysed until PS30 (Fig 2B). A lower dose, 10 µg, gave a correspondingly weaker effect on splicing (Fig 1B; Supporting Information Fig S1C).

**Figure 3:**
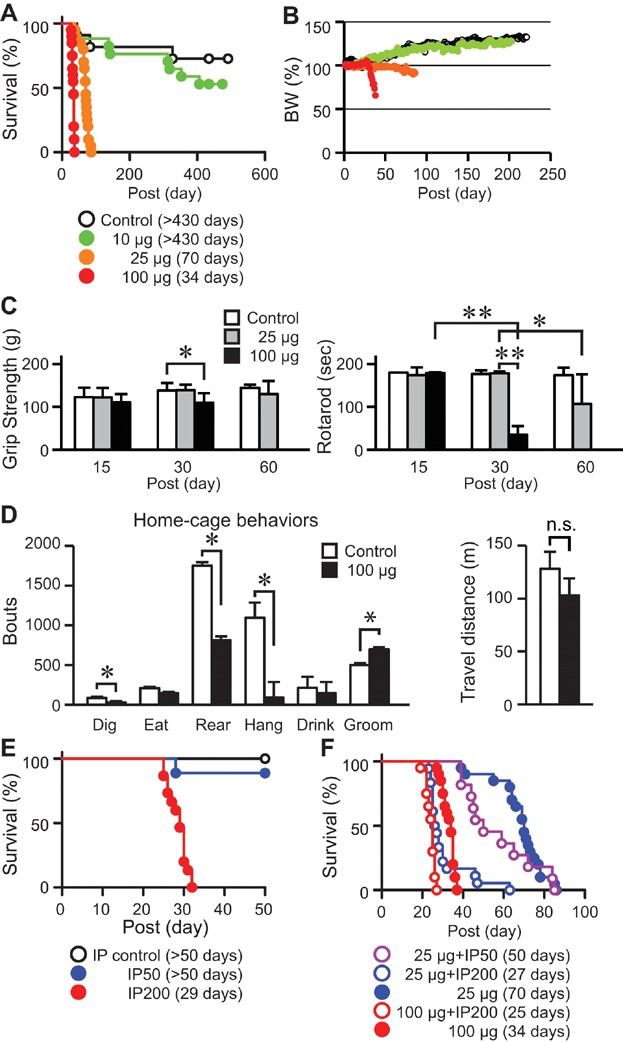
ASO-induction of SMA-like symptoms The median survival days after the first injection are given in parentheses for each group. 10, 25 or 100 µg: the dose of ICV-injected ASO-20-37. IP50 or IP200: IP injection of 50 or 200 mg/kg/day ASO-20-37, respectively. Twenty-five micrograms + IP, or 100 µg + IP: combination of ICV and IP injections of ASO-20-37. We analysed data using two-tailed *t* tests, except for multivariate ANOVA for behaviour analysis and the logrank test for survival analysis. Shortened lifespan. Control mice (*n* = 11) and mice injected ICV with 10 µg (*n* = 17), 25 µg (*n* = 20) or 100 µg (*n* = 20) ASO-20-37 were analysed.Decreased body-weight gain. Control mice (*n* = 5) and mice injected ICV with 10 µg (*n* = 11), 25 µg (*n* = 11) or 100 µg (*n* = 13) ASO-20-37 were analysed.Progressive motor dysfunction. Grip strength (*n* = 10. *p = 0.0062) and rotarod task (*n* = 10. *p = 0.0089; **p < 0.0001) were measured at PS15, 30 or 60.Video-based analysis of home-cage behaviours and travel distance at PS28 (*n* = 8. *p < 0.05; n.s., *p* = 0.5120).IP injection of 200 mg/kg/day ASO-20-37 resulted in reduced lifespan (*n* = 15. *p* < 0.0001), but 50 mg/kg/day ASO did not (*n* = 9. *p* = 0.2918). IP control: IP injection of 200 mg/kg/day control ASO (*n* = 10).IP injection of 50 mg/kg/day in addition to ICV injection of 25 µg (*n* = 11) or 200 mg/kg/day in addition to ICV injection of 25 µg (*n* = 18) or 100 µg (*n* = 20) further reduced survival, compared with the ICV injection alone (*n* = 20).

We previously found that *SMN2* mis-splicing is exacerbated during end-stage SMA, partly as a result of malnutrition (Sahashi et al, 2012). In mice treated with 100 µg ASO, when body weight began to decline at PS30 (Fig 3B), both the levels of exon 7 inclusion and overall SMN expression were further suppressed in the spinal cord, despite an apparent reduction in ASO uptake/accumulation in the CNS (Figs 1D and 2A; Supporting Information Fig S2B). *SMN2* mis-splicing was also exacerbated in the liver and muscle, even though there was only minimal ASO uptake in these tissues (Fig 2A; Supporting Information Fig S2B). Splicing suppression was also observed in late-stage mice that had been injected with 25 µg ASO, but not in mice that were injected with 10 µg ASO (Fig 2B). Simultaneous skipping of exons 5 and 7 in the CNS also increased in late-stage mice injected with 100 µg ASO, and in the spinal cord and liver in the case of mice injected with 25 µg ASO (Fig 2A and B). These observations are consistent with indirect inhibition of *SMN2* splicing in response to ASO treatment, during late-stage disease (Sahashi et al, 2012).

We recently showed that SMN restoration in peripheral tissues is necessary for efficient phenotypic rescue in a severe-SMA mouse model, implying a strong peripheral contribution to SMA pathogenesis (Hua et al, 2011). To study this further in adult mice, we systemically administered ASO-20-37 or control ASO by intraperitoneal (IP) injection, and analysed the resulting *SMN2* splicing patterns in tissues. IP injection of 200 mg/kg/day ASO for 2 consecutive days in 2-month-old mice inhibited *SMN2* splicing markedly in the liver, slightly in the heart and muscle and had no effect in the spinal cord at PS7 (Fig 2C), whereas the control ASO had no effect on splicing in the liver at either PS7 or PS14 (Supporting Information Fig S1A). To establish a dose-dependence, we also injected 20 or 50 mg/kg/day ASO for 2 days. Both doses gave similar effects on splicing in the liver at PS7 or at PS30, and the effects were smaller than with the larger dose of 200 mg/kg/day (Fig 2D).

### Phenotypic effects of exon-skipping ASO

We next studied ASO-20-37's effect on the phenotype of adult *SMN2*-transgenic mice. ICV injection of 25 or 100 µg ASO in 2-month-old mice strikingly shortened their lifespan (median survival: 70 and 34 days post-surgery, respectively; *p* < 0.0001), whereas 10 µg ASO did not (median survival >434 days post-surgery; *p* = 0.8195; Fig 3A). There was also a dose-dependent inhibition of body weight gain, even though the mice were well fed (Fig 3B; Supporting Information Fig S3A). 25 µg ASO-treated mice also displayed kyphosis, a characteristic of SMA (Supporting Information Fig S3A).

In mice injected with 100 µg ASO, body weight and locomotor activity decreased notably after PS30. We observed gait disturbance only late, ∼PS30, after which it progressed further. Some mice were eventually unable to rear themselves, and displayed facial and paw oedema, culminating in death. Other mice died suddenly, before presenting severe paralysis. Serial grip-strength and rotarod tests showed late-onset motor dysfunction (Fig 3C). Automated analysis of home-cage behaviours (Steele et al, 2007) at PS28 revealed significant decreases in digging, hanging and rearing, reflecting limb-muscle weakness (Fig 3D). However, the distance travelled during the recording was not significantly decreased at PS28 (Fig 3D).

Mice injected with 25 µg ASO also showed gait disturbance, typically evident after PS60, and late-onset motor dysfunction was demonstrated by rotarod tests, but not by grip-strength tests (Fig 3C). Although injecting 10 µg ASO reduced exon 7 inclusion to at most 60% of the control level in the spinal cord (Fig 2B), the mice gained weight normally (Fig 3A) and did not have overt motor deficits in walking, rearing and hanging. We did not observe declines in grip strength or rotarod performance at PS120 (Supporting Information Fig S3D). These results demonstrate that ICV injection of ASO-20-37 elicited SMA-like motor dysfunction, growth impairment and shortened lifespan in a dose-dependent manner, even in adult mice, similar to what we previously observed in neonatal mice (Sahashi et al, 2012).

IP injection of 50 mg/kg/day ASO-20-37 for 2 days in adult SMA transgenic mice had no effect on survival (*p* = 0.2918; Fig 3E). However, IP injection of ASO-20-37—but not the control ASO—at a higher dose, 200 mg/kg/day, shortened the lifespan (median survival 29 days post-surgery; *p* < 0.0001; Fig 3E). The body weight began to drop around PS20 (Supporting Information Fig S3B). Locomotor activity also decreased, but the grip-strength or rotarod tasks showed no decline at PS21 (Supporting Information Fig S3C) and no overt paralysis was observed even during end-stage disease. In addition, neither ICV injection of 100 µg nor IP injection of 200 mg/kg/day ASO-20-37 affected normal adult mice with an intact murine *Smn* gene, ruling out off-target effects. Moreover, we observed no impairment in body weight gain for 3–4 months post-surgery in these control mice, and no premature death or overt motor dysfunction during at least 1 year post-surgery.

ICV or IP injection of ASO-20-37 exclusively inhibited *SMN2* splicing in the CNS or peripheral tissues, respectively (Fig 2A and C). Next, we combined ICV and IP injections of ASO-20-37, to elicit more ubiquitous *SMN2* mis-splicing. IP injection of 50 or 200 mg/kg/day ASO for 2 days at PS1 and PS2, after PS0 ICV injection of 25 µg ASO, further reduced survival by ∼30 or ∼60%, respectively, compared with the ICV injection alone (median survival 50 or 27 days post-surgery; *p* = 0.2224 and *p* < 0.0001, respectively). IP injection of 200 mg/kg/day after ICV injection of 100 µg ASO also reduced survival by ∼25% (median survival 25 days post-surgery; *p* < 0.0001; Fig 3F). These results are again consistent with the contribution of peripheral tissues to SMA pathogenesis. The possibility of a direct effect of IP-injected ASO in the CNS, through retrograde axonal transport of ASO to spinal-cord neurons (Crooke, 2007), cannot be completely excluded, although the effect on *SMN2* splicing in the spinal cord was similar between ICV injection of 25 µg ASO with and without IP injection of 200 mg/kg/day ASO (Supporting Information Fig S1D).

### Supportive SMA features

#### Motor neuron pathology

**Figure 4:**
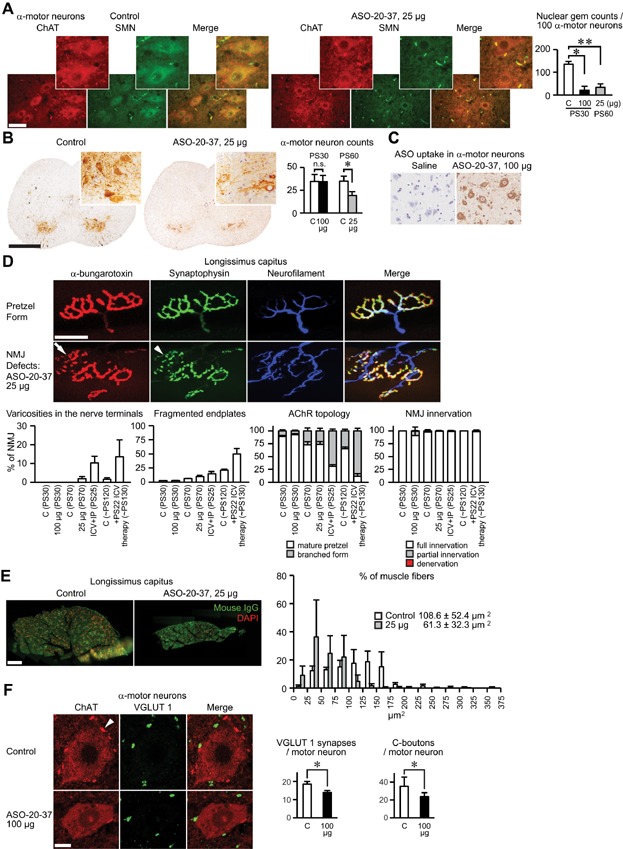
ASO-induced histopathology in motor units For (A–F), C: 100 µg control ASO, 5 µl saline, 25 or 100 µg ASO-20-37 was ICV-injected. We analysed data using two-tailed *t* tests. Decreased nuclear gems. (Left) Dual ChAT and SMN staining of α-motor neurons in PS60 lumbar spinal cord. α-Motor neurons are also displayed at higher magnification in the insets. Scale bar: 50 µm. (Right) Nuclear gem counts in α-motor neurons (*n* = 3. *p = 0.0031; **p = 0.0021).α-Motor neuron loss. (Left) ChAT staining of α-motor neurons in PS60 lumbar spinal cord. Scale bar: 500 µm. (Right) α-Motor neuron counts (*n* = 6. *p = 0.0005; n.s., *p* = 0.9620). (A and B) also show motor-neuron shrinkage in ASO-20-37-treated mice.ASO uptake in PS7 lumbar spinal cord cells, including α-motor neurons.NMJ defects in longissimus capitus (LC) muscle. (Top) NMJs were labelled with α-bungarotoxin (red) for AChRs, anti-synaptophysin (green) for nerve terminals and anti-neurofilament (blue) for nerves. NMJs with a mature pretzel form of AChRs, and NMJ defects of varicosities in the nerve terminals (arrowhead) and fragmented endplates (arrow) are shown. Scale bar: 40 µm. (Bottom) Percentages of varicosities in the nerve terminals, fragmented endplates, AChR topology, and NMJ innervation were quantified in mice treated with: ICV injection of 100 µg control ASO (C) at PS30 (*n* = 3), PS70 (*n* = 4) or PS ∼ 120 (*n* = 3); ICV injection of ASO-20-37 (25 or 100 µg) at PS70 or PS30 (*n* = 4), respectively; ICV injection of 100 µg plus IP injection of 200 mg/kg/day ASO-20-37 (ICV + IP), at PS25 (*n* = 3); or ICV injections of 100 µg ASO-20-37 plus PS22 100 µg ASO-10-27 (+PS22 ICV therapy), at ∼PS130 (*n* = 3).Decreased muscle fibre sizes. (Left) PS70 LC muscle labelled with anti-mouse IgG (green) and DAPI (red). Scale bar: 200 µm. (Right) Distribution of fibre sizes (*n* = 3).Reduced central synapses onto motor neurons. (Left) VGLUT 1 (green) and ChAT (red) staining of nerve terminals and α-motor neurons, respectively, in PS30 spinal cord. Arrowhead represents C-boutons. Scale bar: 10 µm. (Right) The number of VGLUT 1-positive synaptic boutons or C-boutons per motor neuron is shown (*n* = 3. *p = 0.0096 and 0.0010, respectively). The mean value ± standard deviation is shown for each group.

Progressive degeneration of α-motor neurons is a pathological hallmark of SMA (Lunn & Wang, 2008), and indeed, we observed motor-neuron pathology in ICV-injected mice. In accordance with efficient splicing inhibition in the spinal cord (Fig 2A), we observed a marked decrease in the number of SMN-positive nuclear gems—which correlates directly with functional SMN level, and inversely with disease severity (Coovert et al, 1997) in spinal α-motor neurons after ICV injection of 100 or 25 µg ASO (Fig 4A). A significant (>40%) reduction in α-motor-neuron number and shrinkage of motor neurons, indicative of degeneration, were evident in L1–2 spinal cord of mice treated with the lower dose of 25 µg that results in longer survival, when assayed at PS60, but not in mice treated with 100 µg and assayed at PS30 (Fig 4A and B). IHC showed predominant ASO uptake/accumulation in the CNS, including α-motor neurons, rather than in peripheral tissues, in mice ICV-injected with 100 µg ASO at PS7, though the ASO levels declined by PS30 (Fig 4C; Supporting Information Fig S2A and B). Taken together, these results suggest that in the context of ASO-induced SMN deficiency, a rapid disease course in adult mice does not allow sufficient time for motor-neuron degeneration, which proceeds at a lower rate.

#### Muscle histology

We observed little or no cellular ASO uptake in peripheral tissues, such as liver, heart, lung and muscle of mice treated with ICV injection of 100 µg ASO-20-37, at both PS7 and PS30, although we could detect some ASO in liver sinusoids (Supporting Information Fig S2A and B).

Groups of atrophic fibres associated with hypertrophic fibres are typically observed in severe-SMA mice (Hsieh-Li et al, 2000). However, in mice ICV-injected with 25 µg ASO at PS70 or with 100 µg ASO at PS30 (Supporting Information Fig S4), which showed gait disturbance, quadriceps or tibialis anterior sections stained with H&E, acetate non-specific esterase for analysis of denervated fibres or NADH dehydrogenase for analysis of muscle fibre type, showed no SMA-like pathology of fibre-type grouping or atrophy. In light of the marked SMN deficiency restricted to the CNS, these mice may be in a pre-pathological stage in the context of a rapid disease course after induction (Dubowitz & Sewry, 2007), or their phenotype may correspond to late-onset muscle pathology at the adult stages. Alternatively, the limited effects of ASO on *SMN2* mis-splicing in muscle (Fig 2A) may provide a plausible explanation for the lack of overt muscle and heart phenotypes (see below) in the ICV-injected mice.

#### NMJ pathology

NMJ defects, including denervation and delayed maturation, have been described in severe, early-onset SMA (Kariya et al, 2008; Kong et al, 2009; Lee et al, 2011; Ling et al, 2010, 2012; Murray et al, 2008). We first assessed NMJ structure in the vulnerable muscle longissimus capitus (LC) (Ling et al, 2012) in mice that were ICV-injected with 25 or 100 µg ASO, at late-stage PS70 or PS30, respectively. Immunofluorescence staining of NMJs in mice injected with 25 µg ASO, but not with 100 µg ASO, showed abnormal structures in the form of varicosities in the nerve terminals and fragmentation of the endplates (Fig 4D), similar to those reported in aged, damaged, regenerating or diseased muscles in which NMJs undergo extensive remodelling (Couteaux et al, 1988; Duchen et al, 1974; Li & Thompson, 2011; Li et al, 2011; Lyons & Slater, 1991). However, the differences were not statistically significant, probably because of the small sample size (*n* = 3. *p* = 0.1888 and 0.0780, respectively). Postsynaptic acetylcholine receptor (AChR) topology assessed by counting the proportion of mature-pretzel or branched forms (Kummer et al, 2004) was not significantly affected at either ASO dose. In addition, almost all NMJs were fully innervated at both doses, and we did not observe any effects of the presynaptic change, such as denervation atrophy, on postsynaptic LC muscle at PS70 in mice injected with 25 µg ASO (Fig 4D and E). However, the LC muscle of these mice revealed a >40% reduction in muscle fibre area, potentially attributable to the smaller mouse size (Alnaqeeb & Goldspink, 1987; Layman et al, 1980).

To analyse the contribution of peripheral *SMN2* mis-splicing to NMJ structure, we also analysed the mice treated with the combination of ICV injection of 100 µg and IP injection of 200 mg/kg/day ASO-20-37 at PS25, when gait disturbance was observed. We observed a significantly higher incidence of varicosities in the nerve terminals, fragmented endplates and branched forms of AChR (*n* = 3. *p* = 0.0423, 0.0400 and 0.0015, respectively; Fig 4D), although, similar to the ICV injection alone, the combined treatment did not cause significant NMJ denervation. These findings suggest that peripheral *SMN2* mis-splicing additively exacerbates some NMJ pathology.

Considering that morphologically abnormal α-motor neurons and NMJs were observed at the late stages in mice injected with 25 µg ASO, which have relatively slow disease progression, post-developmental NMJ pathology may appear late in the disease course. To address this further, we analysed the NMJs in mice treated with therapeutic ASO-10-27 22 days after ICV injection of ASO-20-37; these mice showed longer disease duration and progressive paralysis (see below). Although denervation was still absent, more extensive NMJ defects were now evident at PS130, which may also be due to muscle regeneration (Fig 4D).

#### Reduction of central synapses on spinal motor neurons

We and others previously showed early defects in central synapses in SMA mice, particularly in the proprioceptive afferent inputs to lumbar motor neurons, which could potentially affect motor-neuron excitability and motor circuitry (Ling et al, 2010; Mentis et al, 2011). To further address the origin of the motor deficits in mice ICV-injected with 100 µg ASO-20-37, we examined the synaptic connectivity between sensory afferents and motor neurons by anti-vesicular glutamate transporter 1 (VGLUT 1) immunostaining for glutamatergic excitatory synapses (Oliveira et al, 2003). We found a significant ∼25% reduction in the number of synaptic boutons on the membranes of motor-neuron soma and proximal dendrites in L1–2 spinal segments at PS30 (Fig 4F).

Cholinergic input from spinal interneurons to motor neurons modulates motor neuron activity (Witts et al, 2013). At C-boutons, a major source of this input, acetylcholine activates muscarinic M2 receptors on motor neurons, and increases the frequency of motor-neuron firing (Miles et al, 2007). Anti-choline acetyltransferase (ChAT) immunostaining also revealed a significant ∼35% reduction in the number of C-boutons on motor neurons (Fig 4F). These results suggest that a partial loss of central synapses could precede motor-neuron degeneration and also play an important role in adult-onset SMA pathogenesis.

#### Heart and liver pathology

**Figure 5:**
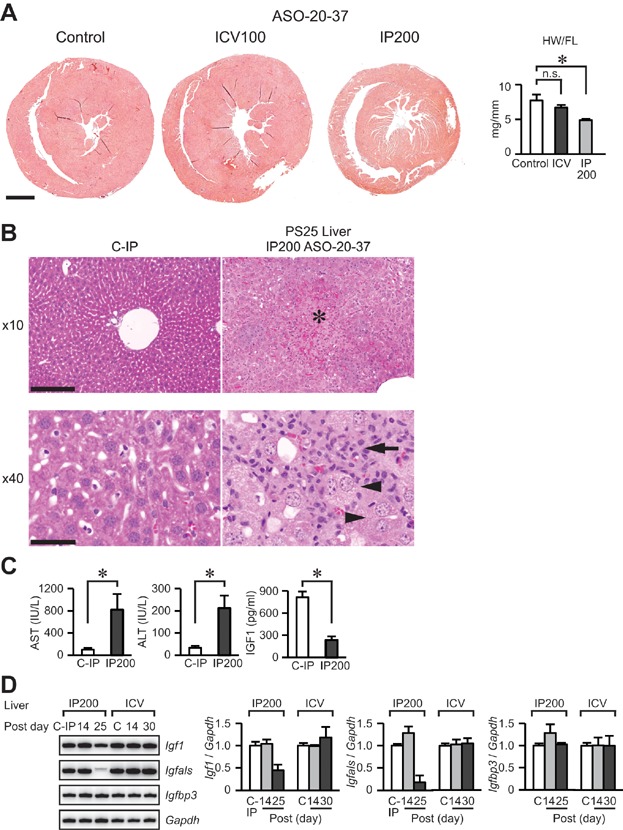
ASO-induced heart and liver pathology We analysed data using two-tailed *t* tests. Reduced cardiac mass at PS25 in mice that received IP injection of 200 mg/kg/day ASO-20-37, but not in mice that received ICV injection of 100 µg ASO-20-37. (Left) H&E staining. Scale bar: 1000 µm. (Right) Cardiac mass normalized to femoral length (*n* = 3. *p = 0.0088; n.s., *p* = 0.1565). HW/FL: cardiac mass/femoral length.Robust liver pathological changes. H&E staining of PS25 liver after IP injection of 200 mg/kg/day ASO-20-37. Hepatocellular necrosis (*), abnormal hepatocytes with large nucleus, oval-cell proliferation (arrowhead) and inflammatory-cell infiltration, particularly neutrophils (arrow), were observed. Scale bar: 200 and 50 µm for 10 and 40× magnification, respectively.Significant elevation in serum AST and ALT levels, markers of liver damage (*n* = 5. *p = 0.0064 and 0.0028, respectively) and decrease in serum IGF1 levels (*n* = 5. *p < 0.0001) at PS30 in the IP ASO-20-37-injected mice.Decline in mRNA levels of hepatic *Igf1* and *Igfals*, but not in *Igfbp3*, at PS25 in the IP-injected mice. In contrast, there was no decline in these mRNAs at PS30 in mice that received ICV injections of 100 µg ASO-20-37. For PS25 *Igfals* in the IP-injected mice, the lower band in each doublet was quantitated. Time-course RT-PCR analysis was performed at PS14, and PS25 or PS30 (*n* = 3). Control or C: ICV injection of 100 µg control ASO; C-IP: IP injection of 200 mg/kg/day control ASO; ICV100: ICV injection of 100 µg ASO-20-37; IP200: IP injection of 200 mg/kg/day ASO-20-37.

Cardiac defects have been reported in severe SMA (Bevan et al, 2010; Heier et al, 2010; Rudnik-Schoneborn et al, 2008). Some of our mice that received ICV injection of 100 µg ASO-20-37 died suddenly, indicating potential cardiac involvement. However, we saw no evidence of cardiomyocyte hypertrophy or atrophy or inflammation, such as cell infiltration and increased interstitial fibrosis in H&E-stained heart sections at PS25 (Fig 5A), prior to the steep physiological decline. We also harvested hearts and weighed them at PS25, but the cardiac mass normalized to femoral length was not significantly altered (Fig 5A). High-resolution echocardiography did not reveal significant alterations in heart rate, contractility, or internal size of the left ventricle (LV), or in the thickness of the interventricular septum (IVS) or LV posterior wall at PS25 (Table 1). In contrast, the normalized heart mass was reduced in the mice treated with IP injection of 200 mg/kg/day ASO-20-37 at PS25, and echocardiography detected a significant LV size reduction and IVS hypertrophy (Fig 5A; Table 1). These changes on echocardiography were confirmed by time-course analysis before and after IP injection of ASO-20-37 (Supporting Information Table S2), which partly reflects the growth impairment. However, the H&E-stained heart sections did not show cellular abnormalities.

**Table 1:** Echocardiographic measurements at PS25 in control or ASO-20-37-treated mice

	Units	Control (*n* = 3)		ICV (*n* = 3)		IP (*n* = 6)	
			
		Average	SD	Average	SD	Average	SD
HR	b/min	523	23	497	14	445	78
IVS; d	mm	0.13	0.03	0.15	0.02	0.19	0.03
LVID; d	mm	3.78	0.08	3.90	0.09	2.93	0.08
LVPW; d	mm	0.99	0.12	0.82	0.08	0.86	0.07
IVS; s	mm	0.13	0.03	0.15	0.03	0.21	0.04
LVID; s	mm	2.12	0.10	2.39	0.06	1.62	0.08
LVPW; s	mm	1.47	0.10	1.29	0.07	1.29	0.09
LV vol; d	µl	61.40	3.00	66.48	3.74	33.86	2.16
LV vol; s	µl	14.95	1.81	20.35	1.41	8.17	0.88
% EF	%	75.76	3.20	69.93	2.18	77.84	2.59
% FS	%	43.95	2.94	39.01	1.83	45.48	2.60
LV mass	mg	67.08	10.07	59.04	6.65	40.58	4.06

HR, heart rate; IVS, interventricular septum; LVID, left ventricular end-diastolic internal dimension; LVPW, left ventricular posterior wall; % EF, ejection fraction: % of (end-diastolic volume − end-systolic volume)/end-diastolic volume; % FS, fractional shortening: % of (end-diastolic dimension − end-systolic dimension)/end-diastolic dimension; d/s, systolic/diastolic; vol, volume.

Control, a single ICV injection of 100 µg control ASO; ICV, a single ICV injection of 100 µg ASO-20-37; IP, 2-day IP injection of 200 mg/kg/day ASO-20-37.

Insulin-like growth factor 1 (IGF1) is a potent trophic factor with therapeutic potential for several motor-neuron diseases, including SMA (Bosch-Marce et al, 2011; Dodge et al, 2010; Palazzolo et al, 2009; Shababi et al, 2011). We previously demonstrated that severe-SMA mice have low levels of serum IGF1, attributable to decreased levels of mRNA encoding hepatic *Igfals* (Hua et al, 2011). The liver, where synthesis of IGFALS mainly occurs, contributes most of the circulating IGF1, whose stability depends on forming a ternary complex with IGFALS and IGF-binding protein 3 (IGFBP3; Baxter & Dai, 1994; Dai & Baxter, 1994; Sjogren et al, 1999). These findings suggested the importance of liver pathogenesis in SMA. Consistent with this notion, IP injection of 200 mg/kg/day ASO-20-37 markedly inhibited *SMN2* splicing in the liver and decreased lifespan (Figs 2C and 3E). Compared with separate ICV or IP injection of ASO-20-37, the combination of ICV and IP injections further reduced lifespan (Fig 3E and F). These results suggest that the ASO-mediated induction of liver pathology may additively contribute to the poor prognosis.

To further address this supposition, we assessed liver histology, focusing on late-stage disease. Remarkably, H&E-stained liver sections at PS25, from the mice treated with IP injection of 200 mg/kg/day, showed prominent multifocal hepatocellular necrosis, degeneration, many enlarged hepatocytes with large nuclei—indicative of mitotic defects—and oval-cell proliferation—indicative of regeneration—accompanied by a massive influx of inflammatory cells (Fig 5B). With the lower-dose of 50 mg/kg/day, we observed milder but significant liver histopathology at PS25. There were no significant pathological changes in the liver at PS14 in these mice, at PS30 in SMA transgenic mice treated with IP injection of 200 mg/kg/day control ASO (Fig 5B), or in normal (*Smn*^+/+^) mice treated with IP injection of 200 mg/kg/day ASO-20-37, or even during end-stage disease at PS35 in the mice injected ICV with 100 µg ASO-20-37. These findings rule out the possibility of chemistry-related toxic effects in the liver of SMA mice. The IP-injected transgenic mice had marked elevation of serum aspartate aminotransferase (AST) and alanine aminotransferase (ALT), which reflect liver damage, and, similarly to severe-SMA mice, they showed a significant decline in serum IGF1 levels at PS30 (Fig 5C). Time-course RT-PCR analysis of liver mRNA from IP-injected mice showed significant reductions at PS25 in the mRNAs encoding *Igf1* and especially *Igfals*, but not *Igfbp3*; in contrast, there was no such reduction in the liver mRNAs from ICV-injected mice at PS30. The *Igf1* and *Igfals* mRNA levels did not decline at PS14 in the IP-injected mice (Fig 5D). These results indicate marked liver pathogenesis with consequently decreased levels of circulating IGF1, caused by hepatic SMN deficiency at the advanced-disease stages in this adult-onset SMA context.

### Amelioration of SMA-like symptoms by a therapeutic ASO

**Figure 6:**
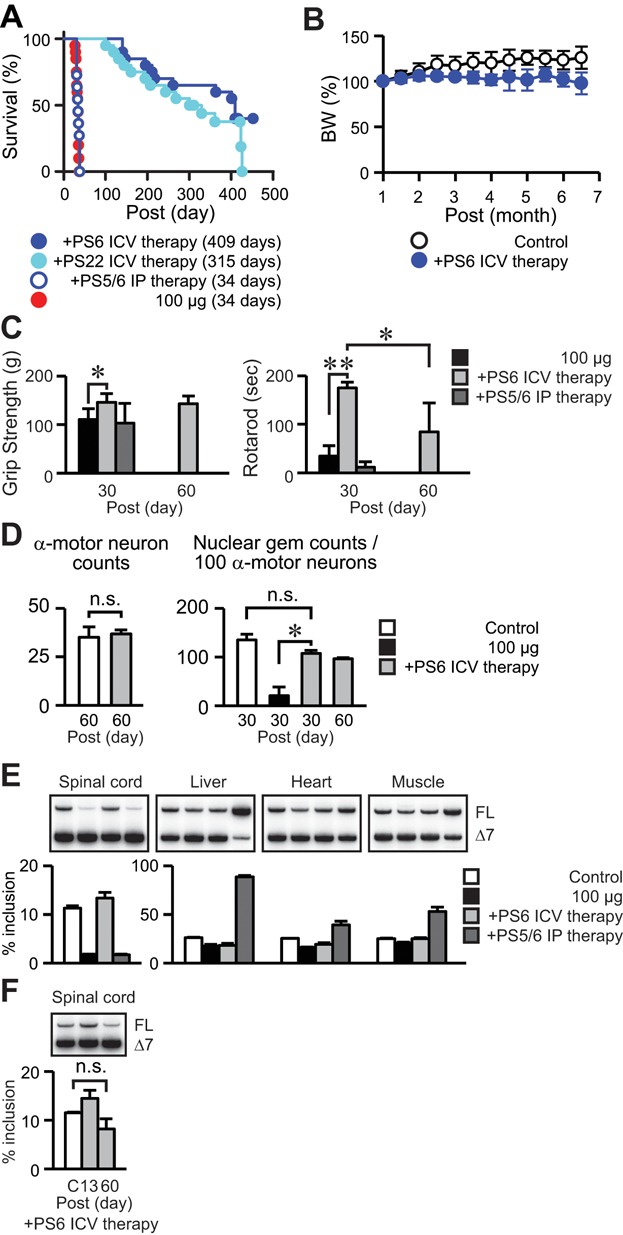
Phenotypic amelioration of ASO-20-37-treated mice by therapeutic ASO C: ICV injection of 100 µg control ASO; 100 µg: ICV injection of 100 µg ASO-20-37; +PS6 or PS22 ICV therapy: ICV injections of 100 µg ASO-20-37 at PS0 and ICV injection of 100 µg ASO-10-27 at PS6 or PS22, respectively; +PS5/6 IP therapy: ICV injection of 100 µg ASO-20-37 at PS0 and IP injections of 200 mg/kg/day ASO-10-27 at PS5 and PS6. We analysed data using two-tailed *t* tests, except for the logrank test for survival analysis. Extended survival. Mice without therapy, with ICV injection of ASO-10-27 (*n* = 20), and with IP injection of ASO-10-27 (*n* = 11) were analysed. The median survival days after the first injection are given in parentheses for each group.Retained body weight. Control mice (*n* = 5) and mice with ICV injection of ASO-10-27 (*n* = 10) were analysed.Improved motor function. Grip strength (*n* = 10. *p = 0.0014) and rotarod task (*n* = 10. *p = 0.0048; **p < 0.0001) were measured at PS30 or 60.Retained α-motor neurons and nuclear gems by ICV injection of ASO-10-27. (Left) α-Motor neuron counts in PS60 lumbar spinal cord (control, *n* = 6. ASO-10-27, *n* = 4. n.s., *p* = 0.5244). (Right) Nuclear gem counts in α-motor neurons in PS30 or PS60 lumbar spinal cord (*n* = 3. *p = 0.0146; n.s., *p* = 0.0689).Restored *SMN2* exon 7 splicing in PS13 tissues (*n* = 3).Retained splicing effect of ICV-injected ASO-10-27 at PS60 (*n* = 3. n.s., *p* = 0.1486).

Therapeutic ASO-10-27 complementary to positions +10 to +27 in *SMN2* intron 7 (Supporting Information Table S1)—non-overlapping with the ASO-20-37-binding site in exon 7—blocks a splicing silencer element (ISS-N1; Singh et al, 2006) and can efficiently increase exon 7 inclusion (Hua et al, 2008; Singh et al, 2006). This ASO, when administered through neonatal ICV and/or systemic injection, efficiently ameliorates the phenotypes of several SMA mouse strains, as well as of our ASO-20-37-induced SMA-phenocopy mice (Hua et al, 2010, 2011; Passini et al, 2011; Sahashi et al, 2012). Here we tested this therapeutic ASO in the adult-onset SMA-phenocopy mice. After ICV injection of 100 µg ASO-20-37 in 2-month-old mice, we administered ASO-10-27 by either ICV injection of 100 µg at PS6 or PS22, or by IP injection of 200 mg/kg/day at both PS5 and PS6. At PS5, we already observed pathology in the form of impaired weight gain (*p* = 0.0003; Fig 3B). In the context of the SMA mice with a lifespan of ∼1-month post-surgery (Fig 3A), ICV injection of ASO-10-27 markedly extended lifespan, even when administered at PS22, although early PS6 treatment was more beneficial (median survival, PS6 and PS22 ICV treatment: 409 and 314.5 days, respectively; *p* < 0.0001; Figs 2A and 6A). The PS6 treatment resulted in sustained body weight (Fig 6B), delayed the onset of motor dysfunction—as shown by grip-strength and rotarod tests (Fig 6C)—and preserved α-motor neuron counts and nuclear-gem number in α-motor neurons (Fig 6D). However, the mice were smaller and thinner than the control mice, and had slowly progressive motor dysfunction and locomotor decline, finally resulting in nearly full paralysis. This terminal state of motor deficits could be partly due to the more severe NMJ defects (Fig 4D).

In contrast, IP injection of ASO-10-27 did not improve survival (median survival 34 days; *p* = 0.2393; Fig 6A). RT-PCR at PS13 showed that ICV injection of ASO-10-27 at PS6 restored *SMN2* splicing in the spinal cord, but had little or no effect on *SMN2* splicing in the liver, heart and muscle. In contrast, IP injection of ASO-10-27 had no splicing effect in the spinal cord, but a significant effect in peripheral tissues (Fig 6E). At PS60, the splicing effect of the ICV-injected therapeutic ASO declined, but remained significant in the spinal cord (Fig 6F).

To further analyse peripheral therapeutic effects, we administered 200 mg/kg/day ASO-10-27 IP 14 and 15 days after IP injection of 200 mg/kg/day ASO-20-37 for 2 days. Even this late treatment showed significant rescue of the mouse lifespan and body weight (Supporting Information Fig S5A and B). With the resulting ameliorated *SMN2* splicing, there was only mild hepatocellular necrosis and inflammatory-cell infiltration in PS30 liver, without an increase in serum AST and ALT levels, a decrease in serum IGF1 levels, and hepatic mRNA levels of *Igf1* and *Igfals* (Supporting Information Fig S5C–F), which clearly demonstrates the therapeutic effects of ASO-10-27 on hepatic pathology.

Taken together, these results indicate that, even after development, the extent of exon 7 skipping in the CNS can be the main determinant of the elicited SMA-like motor symptoms, as is the case during neonatal mouse development (Sahashi et al, 2012). The results also support ASO-10-27 as a drug candidate for adult-onset SMA. The earlier treatment has a better therapeutic outcome, but even delayed treatment appears to be effective.

## DISCUSSION

Severe type I-II SMA patients show symptoms during the newborn and early-infant periods, suggesting a potential developmental component of SMA pathogenesis. On the other hand, many type II–III SMA patients reach adulthood, and there are also adult-onset type IV SMA patients. The pathological impact of post-developmental SMN deficiency remains unclear, which prompted us to address the spatiotemporal requirements for SMN in adult mice.

We have shown that exon-skipping ASOs can be used to phenocopy certain diseases by gene-targeted modulation of alternative splicing. By inhibiting *SMN2* exon 7 inclusion with an appropriate ASO in neonatal (Sahashi et al, 2012) or adult (this study) transgenic mice, we elicited phenotypes resembling canonical SMA. We first used ICV delivery of exon-skipping ASO-20-37 in adult SMA mice with four copies of an *SMN2* transgene, a method whose effectiveness we previously showed in neonates of the same strain (Sahashi et al, 2012). A single injection had dose-dependent effects on *SMN2* splicing in the CNS, correlating with lifespan, growth and motor-function phenotypes (Figs 1 and 3). Notably, compared with our previous finding that neonatal ICV injection causes progressive paralysis, beginning after 2 weeks (Sahashi et al, 2012), in the present study, abnormal gait or rearing became apparent only 1 month after ASO injection. One hundred micrograms of ASO-20-37 robustly inhibited *SMN2* exon 7 splicing as early as PS7 (Fig 1), revealing a time lag between SMN depletion and the motor-phenotype onset. Importantly, our results suggest that the late-onset paralysis reflects a tolerance of mature motor units against SMN deficiency (discussed below), although it is accompanied by motor-unit pathologies. IP injection failed to elicit overt paralysis (Supporting Information Fig S3), even during end-stage disease, revealing that motor dysfunction is not merely secondary to end-stage disease conditions. However, adult-onset paralysis in type IV SMA also reflects compensatory sprouting of nerve terminals, which is not seen in our mice, likely because of the rapid disease course (Dubowitz & Sewry, 2007) as well as the small reduction in muscle SMN.

ICV injection of 10 µg ASO, which reduced the proportion of exon 7 inclusion in the spinal cord of mice with four *SMN2* copies by at most ∼60%, failed to elicit overt phenotypes (Figs 2 and 3). This implies that a threshold for the adult-onset phenotypes would be less than two transgene copies, in this strain background. Another explanation is that ICV injection of 10 µg ASO might elicit only minimal phenotypes that are hardly detectable, or might not sustain *SMN2* mis-splicing to result in significant phenotypic defects, although mis-splicing was evident until at least PS120 (Fig 2). Considering the severity of SMA in neonatal mice with two *SMN2* copies, our results indicate that the SMN levels required for healthy adult mice, especially in the CNS, are less than for neonatal mice. This notion is partly supported by our previous study in severe-SMA mice rescued by therapeutic ASO-10-27: high levels of correction of *SMN2* splicing are not persistently required, at least in the liver, for long-term survival (Hua et al, 2011). Also, for *SMN2*, a minimum of two copies are thought to be necessary after 1 month postnatally in SMA Δ7 mice (Le et al, 2011), which in turn indicates a lower post-developmental demand for SMN.

ICV injection of ASO-20-37 resulted in a marked decrease in the number of SMN-containing nuclear gems—an indicator of SMN protein abundance (Coovert et al, 1997) in α-motor neurons (Fig 4), which is expected to cause neuronal dysfunction. A dose of 25 µg ASO caused α-motor neuron loss and shrinkage, by 2 months post-injection (Fig 4). A dose of 100 µg ASO did not cause such changes by 1 month post-injection, although we observed a loss of glutamatergic or cholinergic excitatory synapses onto motor neurons, which should affect neuronal circuits and in turn motor function (Fig 4). Glutamatergic synaptic pathology occurs early in the course of disease (Ling et al, 2010; Mentis et al, 2011), and cholinergic synaptic pathology may also play a role in the motor deficits (Zagoraiou et al, 2009). Considering that neonatal ICV injection of the ASO leads to significant motor-neuron loss at P30 (Sahashi et al, 2012), morphological defects in α-motor neurons and also NMJs (Fig 4, see below), once their maturation is complete, might only occur to a limited extent and in a delayed manner in the context of SMN deficiency, which may account for the lack of muscle denervation (Fig 4; Supporting Information Fig S4).

ICV injection of ASO-20-37 elicited motor-neuron pathology, and especially when combined with IP injection, it led to NMJ pathology, as well as overt motor-function deficits. These findings reveal that SMN function is required for α-motor neuron and NMJ maintenance even after embryonic development, although the threshold levels of SMN appear to be lower than for neonate mice, meaning that mature motor units can tolerate relatively low SMN levels.

Whether SMN deficiency in adulthood affects NMJ function has not been determined. Removal of SMN at 1 month postnatally in the SMA Δ7 mouse results in no impairment in synaptic transmission at NMJs, 1 month later, at a time when the mice are dying (Le et al, 2011). Thus, it is unlikely that neuromuscular transmission was impaired in the mice in which we ICV-injected 100 µg of ASO-20-37, which also die in 1 month. Instead, pathology in the motor-neuron circuitry, as reported previously (Ling et al, 2010; Mentis et al, 2011; Park et al, 2010b), could explain the motor phenotypes in our model.

A higher proportion of abnormal NMJ structures were observed with a longer disease course (Fig 4), indicating a temporal requirement for the defects to arise in adults with SMN deficiency. The delayed-onset motor neuron dysfunction may also affect efferent nerve terminals and endplates (Gogliotti et al, 2012; Park et al, 2010b). In contrast, our finding that combined ICV and IP injection of ASO exacerbated NMJ defects suggests additional NMJ pathology with peripheral SMN deficiency (Fig 4). We demonstrate that relatively mild NMJ pathology could be seen in adult-onset SMA mice, in contrast to early-onset SMA mice (Kariya et al, 2008; Kong et al, 2009; Lee et al, 2011; Ling et al, 2010, 2012; Murray et al, 2008; Park et al, 2010a; Sahashi et al, 2012) whose endplate maturity or NMJ innervation is affected. These motor-unit pathologies in mouse models imply that SMN is especially important during development, which in turn suggests that the pathogenesis of SMA has important developmental components.

Because of the inability of MOE ASOs to cross the mature BBB, systemic IP injection of ASO-20-37 inhibited *SMN2* splicing only in peripheral tissues, especially liver. However, we observed only limited splicing effects of ASO-20-37 in muscle and heart (Fig 2), presumably reflecting the limited ASO biodistribution in these tissues and/or different levels of *trans*-acting factors that regulate *SMN2* splicing. As IP administration affected survival and unveiled some heart and liver pathology (Figs 3 and 5), analysing the effect of enhanced, persistent *SMN2* mis-splicing in muscle or heart may further help to address post-developmental pathogenesis, especially using different administration routes and/or different ASOs.

Echocardiography before end-stage disease showed no significant defects in mice injected ICV with ASO-20-37 to substantially inhibit *SMN2* splicing in the CNS (Table 1; Fig 2). However, despite the absence of a significant decline in heart rate, our finding that some mice suddenly died suggests a possible end-stage involvement of severe central autonomic dysfunction, which has been implicated in cardiac dysfunction in severe SMA (Bevan et al, 2010; Heier et al, 2010). In contrast to ICV injection of ASO-20-37, IP injection induced cardiac hypertrophy and a reduction in cardiac mass (Fig 5; Table 1; Supporting Information Table S2). Because there was no cellular pathology, impairments in cardiac growth should be considered in this case. SMN deficiency in other tissues could also contribute to cardiac complications. In particular, liver dysfunction might induce cardiomyopathy, in part by hyperdynamic circulation (Ma & Lee, 1996; Moller & Henriksen, 2010), and/or an accompanying decrease in circulating IGF1 levels (see below) might affect cardiac function (Juul et al, 2002; Vasan et al, 2003).

We identified pronounced liver pathology in mice that received IP injection of ASO-20-37, but not in mice that received ICV injection. Accordingly, in the IP-injected mice, the mRNA levels of hepatic *Igf1* and *Igfals* decreased (Fig 5). Most of the circulating IGF1 is in the form of stable complexes with IGFALS and IGFBP3 (Baxter & Dai, 1994). Therefore, the reduction in IGF1 and IGFALS would account for the observed low levels of circulating IGF1 (Fig 5; Sjogren et al, 1999). These liver pathologies must be a consequence of hepatic *SMN2* mis-splicing, because they were prevented by subsequent IP injection of splicing-correcting ASO-10-27 (Supporting Information Fig S5). They may contribute to SMA progression (Hua et al, 2011), underscoring the importance of SMA pathology in peripheral tissues. Knockout of SMN in mouse liver results in defective liver development, with lack of regeneration (Vitte et al, 2004). In contrast, our approach did not completely suppress *SMN2* splicing at the adult disease stages, and resulted in distinctive liver pathology, including oval cell-mediated regeneration (Figs 2 and 5). The combination of ICV and IP injections of ASO-20-37 further shortened lifespan, compared to either injection alone, emphasizing that hepatic and cardiac complications should be also considered as features of adult SMA, especially at the advanced stages, when *SMN2* mis-splicing is exacerbated (see below; Fig 2; Sahashi et al, 2012).

*SMN2* was further mis-spliced in both the CNS and peripheral tissues in the late-stage mice that were ICV-injected with ASO-20-37 (Fig 2), in a manner that did not correlate with the extent of ASO uptake in various tissues. This finding is indicative of progressive *SMN2* mis-splicing in the context of end-stage SMA (Sahashi et al, 2012). ASO-induced SMN deficiency may trigger a further decrease in *SMN2* splicing through a feedback loop (Jodelka et al, 2010; Ruggiu et al, 2012), on top of which, end-stage-disease conditions, such as nutritional deficiency and hypoxia may cause widespread splicing alterations, including in *SMN2* (Bebee et al, 2012; Sahashi et al, 2012), each of which may potentially accelerate SMA progression. Thus, controlling stress conditions caused by SMA progression and/or other complications may be very important for its prognosis.

ASO-20-37, which specifically targets the human *SMN2* transgene, did not affect normal mice with an intact *Smn* gene. We previously showed that neonatal ICV injection of ASO-20-37 phenocopies SMA in *SMN2*-transgenic mice (Sahashi et al, 2012), and here, we similarly recapitulated this phenotype in adult mice of the same strain. The phenotypic amelioration of these ASO-20-37-treated mice by a therapeutic ASO that restores *SMN2* splicing (Fig 6) demonstrates that the SMA-like phenotypes were elicited through *SMN2* mis-splicing, excluding potential off-target effects as contributors to disease onset.

Compared with RNAi-based or antisense-knockdown approaches, our method, which we previously dubbed TSUNAMI (for targeting splicing using negative ASOs to model illness), retains the primary transcript and thus enables testing of therapeutics that correct splicing of the target pre-mRNA (Sahashi et al, 2012). By targeting the human *SMN2* transgene, we were able to use therapeutic ASO-10-27 for splicing-rescue experiments in the SMA-phenocopy mouse model. Its ability to correct splicing in the CNS or peripheral tissues correlated with phenotypic and histological amelioration in ASO-20-37-treated mice (Fig 6; Supporting Information Fig S5).

We reported that neonatal systemic injection of ASO-10-27 efficiently rescues severe-SMA mice (Hua et al, 2011). However, this systemic treatment had no therapeutic benefit in adult mice that were first administered ASO-20-37 by ICV injection (Fig 6). This is similar to what we observed with neonatal mice sequentially treated with these ASOs (Sahashi et al, 2012). As we discussed in that study, the inconsistency in the effect of systemic ASO treatment among these studies may reflect spatial and temporal differences in *SMN2* splicing patterns; here *SMN2* splicing (from four copies of *SMN2*) was predominantly inhibited in the CNS at the adult stages, whereas in severe-SMA mice *SMN2* mis-splicing (from two copies of *SMN2*) is ubiquitous and begins embryonically. Another important difference between neonatal and adult mice is the extent of BBB closure (Stewart & Hayakawa, 1987), which determines whether a systemically administered MOE ASO reaches the CNS. However, the present results do suggest that SMN levels in CNS tissues determine SMA-like motor phenotype at the adult stage, whereas those in both CNS and peripheral tissues correlate with overall prognosis. Our results also underscore a potentially wider therapeutic time window for adult SMA than previously believed based on studies in severe-SMA mice (Foust et al, 2010; Hua et al, 2011; Le et al, 2011; Lutz et al, 2011).

Except for the tail and ear necrosis, our model has an asymptomatic phase before disease onset at the adult stage, as seen in type IV SMA. Although basal SMN levels are below normal, due to the mouse genotype, SMN is further reduced in a tissue-specific manner, by ASO treatment, which is distinct from the situation in SMA patients. The disease progression after onset in our model is more acute, and without chronic compensation. However, here and also in our previous study (Sahashi et al, 2012), TSUNAMI can help to elucidate relevant phenotypes, and our spatial and temporal analyses of SMN's roles provide new insights into SMA pathogenesis and targeted-therapeutic strategies.

Here, we have shown that a single injection of an exon-skipping ASO into the cerebral ventricles phenocopies adult-onset SMA. Compared with neonatal administration, this procedure elicited late-onset motor phenotypes. In adult mice with *Smn*-null background, the SMN levels required for normal CNS function correspond to an *SMN2* copy number less than two, suggesting that only moderate SMN levels are necessary for therapy in adult-onset SMA. The effects of peripheral *SMN2* mis-splicing induced by TSUNAMI highlight the potential importance of specific pathogenesis in the liver and heart. We also showed a ubiquitous exacerbation of *SMN2* mis-splicing during late-stage disease, which likely accelerates disease progression. Finally, our results suggest a broad therapeutic time window for ASO-10-27.

Compared with early-onset SMA, adult-onset SMA may have distinct pathology, as well as therapeutic responses and requirements, perhaps reflecting key differences between development and post-development.

## MATERIALS AND METHODS

### Oligonucleotides

2′-MOE oligonucleotides with a phosphorothioate backbone and all 5-methylcytosines were synthesized and purified as described (Hua et al, 2010). The ASO sequences are listed in Supporting Information Table S1.

### Animals

Mouse protocols were approved by Cold Spring Harbor Laboratory's Institutional Animal Care and Use Committee. SMA transgenic mice (*Smn*^−/−^ *SMN2*^+/+^) with four *SMN2* copies were purchased from The Jackson Laboratory (strain FVB.Cg-Tg(SMN2)2Hung Smn1^tm1Hung^/J). Mice were fed a normal chow diet (PicoLab Rodent Diet 20, LabDiet) and, when required, a nutrient-fortified water gel diet (DietGel Recovery, ClearH2O).

### ICV injection

Two-month-old (∼60 days postnatally) mice were anaesthetised with 2% isoflurane and held by the head in a stereotaxic instrument. Five microliters of each ASO in saline was injected into the right lateral ventricle. The coordinates for injection were: 1 mm lateral from the sagittal suture, 0.2 mm posterior from the bregma and 3 mm deep from the brain surface.

### RNA and protein analyses

For each experimental group, RT-PCR with ^32^P-dCTP was performed with tissues from three mice. Total RNA extraction and RT-PCR were performed to analyse *SMN2*, *Igf1*, *Igfals*, *Igfbp3* and *Gapdh* transcripts, as described (Hua et al, 2008, 2011). The brain cortex, thoracic spinal cord, heart, liver and quadriceps muscle were used. PCR products were analysed as described (Sahashi et al, 2012). The expression of human SMN or α-tubulin protein in the thoracic spinal cord was analysed by Western blotting, as described (Sahashi et al, 2012). Serum IGF1 levels were measured with a Mouse/Rat IGF-I Quantikine ELISA kit (R&D Systems).

### Mouse physiology

The rotarod task and grip-strength test were carried out as described (Sahashi et al, 2012). Mouse behaviour phenotyping was performed using a video-based behaviour recording setup (Steele et al, 2007). Mice entered the recording cages at 4 pm and were continuously recorded through the dark phase (7 pm–7 am). Data were analysed using HomeCageScan software (Clever Sys). The unit for travel distance is metres, and that for behaviours is the number of times (bouts). Transthoracic echocardiography was performed on anaesthetised mice with 1–2% isoflurane using a Vevo 770 ultrasound device (VisualSonics, Canada) with a 30-MHz transducer. The Vevo Measurement and Calculations software package was used to generate left-ventricular measurements and calculations.

**The paper explained**

**PROBLEM:**

Spinal muscular atrophy (SMA), a leading genetic cause of infant mortality, is caused by reduced levels of SMN protein. Patients with severe SMA show symptoms shortly after birth, suggesting a potential developmental component of SMA pathogenesis. However, many SMA patients reach adulthood, and there is also an adult-onset form of SMA. There is a lack of animal models of adult-onset SMA, and the post-developmental components of SMA pathogenesis remain elusive.

**RESULTS:**

We used antisense-oligonucleotide (ASO) technology to disrupt splicing of *SMN2* mRNA, which encodes SMN protein. ASO injection into the CNS of adult *SMN2*-transgenic mice mimicked adult-onset SMA, with a characteristic late-onset motor phenotype. Systemic ASO injection elicited marked liver and heart pathologies, and resulted in decreased levels of circulating IGF1. *SMN2* mis-splicing increased during late-stage disease. Our study also suggests that high SMN levels are not as critical in adults, and there is a broad therapeutic time window for treatment of adult-onset SMA.

**IMPACT:**

In this study, we successfully generated an adult-onset SMA mouse model. Compared with early-onset SMA, adult-onset SMA may have distinct pathology, as well as therapeutic responses and requirements, potentially reflecting important differences between development and post-development. We also report that the adult-SMA model—like the early-onset model—displays late-stage exacerbation of *SMN2* mis-splicing, which likely accelerates disease progression.

### Mouse histology

After staining with ChAT or SMN antibodies, the number of α-motor neurons or nuclear gems was counted as described (Sahashi et al, 2012). ASO uptake in tissues was assessed with antibody against the phosphorothioate backbone. NMJs were immunolabelled, and the proportion of innervated NMJs or the percentage of each AChR topology was quantified as described (Sahashi et al, 2012). After obtaining 80-µm sections from L1–2 spinal segments, the 3rd and 6th sections were immunostained with anti-VGLUT 1 antibody (1:1000, Synaptic Systems) and anti-ChAT antibody (1:100, Chemicon) for synapses and motor neurons, respectively. Z-stacks of images with a 1-µm interval were taken, and the number of synaptic boutons around motor-neuron soma and proximal dendrites was counted. Flash-frozen muscle and formalin-fixed heart and liver were cut for staining. Muscle fibre sizes were quantified using ImageJ software (http://rsb.info.nih.gov/ij). Images were acquired with an Axio Observer.Z1 microscope and an LSM 710 confocal microscope (Carl Zeiss, Germany) for bright-field and immunofluorescence imaging, respectively.

### Statistical analysis

We analysed data using two-tailed *t* tests, and considered *p* values ≤0.05 to be statistically significant. For analysis of home-cage behaviours, Levene's, Kolmogorov–Smirnov and Shapiro–Wilk tests for normality were used. Multivariate ANOVA was used to observe any statistical differences for each behaviour. All tests were performed in the PASW Statistics system (SPSS, Inc.). Kaplan–Meier survival curves were prepared with Prism 5 (GraphPad Software) and statistical significance was calculated with the logrank (Mantel–Cox) test. The various histograms and plots show mean values ± standard deviation.

## For more information

OMIM for Spinal Muscular Atrophy:

http://omim.org/entry/253300

Families of SMA:

http://www.fsma.org

SMA Foundation:

http://www.smafoundation.org

FightSMA:

http://www.fightsma.org

Muscular Dystrophy Association:

http://www.mda.org

## Supplementary material

As a service to our authors and readers, this journal provides supporting information supplied by the authors. Such materials are peer reviewed and may be re-organized for online delivery, but are not copy-edited or typeset. Technical support issues arising from supporting information (other than missing files) should be addressed to the authors.

**Figure S1. Splicing effect of control ASO, therapeutic ASO-10-27 or ASO-20-37.** (A) ICV injection of 100 μg or IP injection of 200 mg/kg/day control ASO did not affect SMN2 splicing in spinal cord or liver, respectively, analysed at PS7 or PS14 (*n* = 3). (B) Time-course analysis of the effect of ICV injection of 100 μg ASO-10-27 on SMN2 splicing in spinal cord (*n* = 3). (C) Compared with PS7, 10 or 25 μg ASO-20-37 further inhibited exon 7 inclusion at PS30 in a dose-dependent manner (*n* = 3). Twenty-five micrograms ASO has a stronger effect than 10 μg. (D) Similar splicing effect in PS7 spinal cord between ICV injection of 25 μg ASO-20-37 with or without additional IP injection of 200 mg/kg/day ASO-20-37 (*n* = 3). FL, Δ7 and Δ5 + Δ7: full-length, exon-7-skipped transcripts and mRNA lacking both exon 5 and exon 7, respectively. S: 5 μl saline, C: 100 μg control ASO; 25 + IP: combination of ICV injection of 25 μg and IP injection of 200 mg/kg/day ASO-20-37.

**Figure S2. ASO uptake in tissues 7 days (A) or 30 days (B) after ICV injection of 100 μg ASO-20-37 (IHC).** ASO is broadly detected in CNS cells, and mainly in liver sinusoids. ASO is only weakly detectable in heart, lung and quadriceps muscle. Compared with PS7, PS30 uptake declines in the CNS. The brain and spinal cord regions indicated by open circles are also displayed at higher magnification in the insets. Scale bar: 1000 and 200 μm for 6 and 40× magnification. Saline: ICV injection of 5 μl saline.

**Figure S3. Phenotypic effect of ASO-20-37.** (A) Mice that receive ICV injection of 25 μg ASO-20-37 are smaller and show kyphosis. (B) Mice begin to lose body weight around 20 days after IP injection of 200 mg/kg/day ASO-20-37. Control mice (*n* = 10) and mice injected with ASO-20-37 (*n* = 12) were analysed. (C) IP injection of ASO-20-37 had no effect on motor function, analysed by grip strength (*n* = 7. n.s., *p* = 0.9122) and rotarod task (*n* = 7. n.s., *p* = 0.3599) measured at PS21. (D) ICV injection of 10 μg ASO-20-37 had no effect on motor function, analysed by grip strength (*n* = 3. n.s., *p* = 0.6601) and rotarod task (*n* = 3. n.s., *p* = 0.4227) measured at PS120. Control: ICV injection of 100 μg control ASO; IP control: IP injection of 200 mg/kg/day control ASO. We analysed data using two-tailed *t* tests.

**Figure S4. Muscle histology.** No overt muscle pathology in quadriceps (top) or tibialis anterior (bottom) is seen at end-stage PS70 in the mice given ICV injection of 25 μg ASO-20-37. H&E, acetate non-specific esterase and NADH dehydrogenase staining. Scale bar: 200 μm.

**Figure S5. Amelioration by systemically administered therapeutic ASO.** Two hundred milligrams per kilogram per day therapeutic ASO-10-27 was injected intraperitoneally 14 and 15 days after IP injection of 200 mg/kg/day ASO-20-37. (A) Extended survival by ASO-10-27. Control mice (*n* = 10), mice without therapy (*n* = 15) and with IP injection of ASO-10-27 (*n* = 10) were analysed. (B) Retained body weight. Control mice (*n* = 10), mice without therapy (*n* = 12) and with IP injection of ASO-10-27 (*n* = 10) were analysed. The median survival days after the first injection of ASO-20-37 are given in parentheses for each group. (C) Increased exon 7 inclusion in SMN2 in P30 liver (*n* = 3). (D) Ameliorated histological change in liver at PS30. There was only mild hepatocellular necrosis with sporadic enlarged cells (arrow), and inflammatory cell infiltration (arrowhead), particularly neutrophils. H&E staining. Scale bar: 200 μm. (E) Retained serum AST and ALT levels (*n* = 5. n.s., *p* = 0.7732 and 0.4442, respectively) and IGF1 levels (*n* = 5. n.s., *p* = 0.1472). (F) Retained mRNA levels of hepatic Igf1 and Igfals at PS30 (*n* = 3). For Igfals in the mice treated with ASO-20-37 only, the lower band in each doublet was quantitated. IP control or IP200 ASO-20-37: IP injection of 200 mg/kg/day control ASO or ASO-20-37 for 2 days, respectively; +PS14/15 IP therapy: IP injection of 200 mg/kg/day ASO-20-37 for 2 days and IP injections of 200 mg/kg/day ASO-10-27 at PS14 and PS15. We analysed data using two-tailed *t* tests, except for the logrank test for survival analysis.

**Table S1.** Sequences of the uniform 2′-MOE ASOs with phosphorothioate backbone and 5-methylcytosines used in this study.

**Table S2.** Echocardiographic measurements before and 25 days after IP injection of control ASO or ASO-20-37.
