# Supplementary material for: Pathological impact of SMN2 mis-splicing in adult SMA mice
Source: EMBO Mol Med. 2013 Sep 9;5(10):1586–601. doi: 10.1002/emmm.201302567 (PMC3799581; doi:10.1002/emmm.201302567)
Supplement: Supplementary file 1 [file emmm0005-1586-SD1.pdf]

EMBO Molecular Medicine

Pathological impact of SMN2 mis-splicing in adult SMA mice

Kentaro Sahashi, Karen K. Y. Ling, Yimin Hua, J. Erby Wilkinson, Tomoki Nomakuchi, Frank Rigo, Gene Hung, David Xu, Ya-Ping Jiang, Richard Z. Lin, Chien-Ping Ko, C. Frank Bennett, and Adrian R. Krainer

Corresponding author: Adrian Krainer, Cold Spring Harbor Laboratory

Review timeline:

Submission date:	29 January 2013
Editorial Decision:	04 March 2013
Additional Author correspondence:	13 May 2013
Additional Editorial Correspondence:	15 May 2013
Revision received	06 July 2013
Editorial Decision:	23 July 2013
Revision received	06 August 2013
Accepted:	09 August 2013

Transaction Report:

(Note: With the exception of the correction of typographical or spelling errors that could be a source of ambiguity, letters and reports are not edited. The original formatting of letters and referee reports may not be reflected in this compilation.)

Editor: Roberto Buccione

1st Editorial Decision 04 March 2013

Thank you for the submission of your manuscript to EMBO Molecular Medicine. We have now received reports form the three Reviewers whom we asked to evaluate your manuscript

You will see that while the Reviewers are generally supportive, they raise important concerns that ultimately question the relevance of your mouse model for human disease. Altogether these concerns prevent us from considering publication at this time. I will not dwell into much detail, as the evaluations are self-explanatory. I would like, however, to highlight some major points.

Reviewers 2 and 3 note while human late onset SMA pathology is caused by a chronic, subtle under-supply of SMN protein that causes a progressive paralysis, in your model, instead, a severe SMN shortage is suddenly induced at old age, which causes severe paralysis and death. Furthermore, all three Reviewers are concerned that there is no report of the other pathological manifestations of human disease such as the involvement of other major organs. Another item of concern is that it remains unclear whether onset of motor dysfunction precedes non-specific effects of excessive SMN depletion. The reviewers also mention a few other critical issues that require your action.

I am sure that you will appreciate how important translation to human disease is for EMBO Molecular Medicine. Therefore, while publication of the paper cannot be considered at this stage, we would be prepared to consider a suitably revised submission, with the understanding that careful further analysis of the mouse model is required along the lines indicated by the Reviewers, with provision of additional experimental data where appropriate.

© EMBO

Please note that it is EMBO Molecular Medicine policy to allow a single round of revision only and that, therefore, acceptance or rejection of the manuscript will depend on the completeness of your responses included in the next, final version of the manuscript.

As you know, EMBO Molecular Medicine has a "scooping protection" policy, whereby similar findings that are published by others during review or revision are not a criterion for rejection. However, I do ask you to get in touch with us after three months if you have not completed your revision, to update us on the status. Please also contact us as soon as possible if similar work is published elsewhere.

I look forward to seeing a revised form of your manuscript as soon as possible.

***** Reviewer's comments *****

Referee #1 (General Remarks):

This manuscript from Sahashi and colleagues reports on an important set of experiments, detailing the consequences for reducing full-length SMN protein levels in adult SMA mice. The authors have utilised robust antisense oligonucleotide approaches to manipulate splicing of the SMN2 gene in vivo, and they have gone to great lengths to demonstrate the accuracy and repeatability of this approach. The study is very well designed and performed, and represents a huge body of work. The main findings of the study, revealing that disruption of SMN splicing in adult mice can be used to model adult-onset forms of SMA and showing that the adult CNS can tolerate lower levels of SMN protein than in neonatal animals, are well supported by the experimental data. There are, however, a few issues that would need to be addressed before I could recommend publication:

Major points

1) My main concern with the study is the lack of information regarding the effect of the ASOs on control, healthy mice. On page 9, the authors report that "neither ICV nor IP injection of ASO-20-37 affected normal mice with an intact murine Smn gene, ruling out off-target effects". These experiments are very important for the interpretation of the results from experiments in SMA mice and need further clarification. What doses were provided to the control mice and what time-points were examined? How was the health of the mice evaluated? Moreover, given that the SMA mice have liver defects (as previously reported by these authors) is it possible that the SMA mice had a lower tolerance to the ASO and therefore lower doses could elicit toxic effects in vivo? To rule out these possibilities, the authors might want to show that high doses of the ASO (extending beyond those used in the SMA model) have no effects on healthy wild-type mice.

2) The authors show in Fig 2C that IP delivery of their ASO had limited effects on splicing in muscle (and to a lesser extent heart). Given that both muscle and heart are thought to be directly affected in SMA, might these results have implications for the severity of the resulting phenotype observed in the SMA mice? For example, the lack of muscle pathology that the authors observe (page 10) could potentially be explained by the lack of changes in full-length protein expression (e.g. muscle pathology might be expected to occur if the ASO successfully targeted muscle), rather than indicating that muscle pathology is only a late event in the disease (as the authors propose).

3) Given the growing body of evidence for the involvement of peripheral tissues in SMA other than the liver (as previously shown by these authors), such as heart, lungs, pancreas, GI tract, bone etc (recently reviewed in PMID: 23228902), did the authors observe any other systemic pathology in the ASO-treated SMA mice? Did they check for splicing effects in any other organs or tissues in the mice treated with ASO via IP delivery?

Minor comments:

1) On page 6 (second last sentence), the authors refer to data from PS7 and PS14 in Fig 1, but only the former is shown.

2) I found the discussion to be a bit too long (just a personal opinion, I acknowledge!). As a result, some of the main messages got rather lost by the end. It may benefit from a bit of judicious pruning.

3) It is unclear what the graphs are actually showing in Fig 2A and Fig 2B. The figure legends need to be amended accordingly.

4) It would be helpful for N numbers and statistical tests to be included in all the figure legends.

Referee #2 (General Remarks):

This manuscript describes the phenotypic effects of lowering SMN expression in adult mice using antisense oligonucleotides (ASOs) that target SMN2 exon 7 splicing. The spatial requirement for SMN is investigated through behavioral and histopathological analysis following ASO injection either in the CNS or in the periphery. Although low SMN levels in either compartment lead to weight loss and lethality, the mechanisms appear remarkably distinct. SMN depletion in the CNS affects motor function, while low SMN levels in the periphery elicit liver and heart pathology but have no effects on the neuromuscular system. As expected, these phenotypes are corrected by injection of a therapeutic ASO that restores SMN expression by promoting exon 7 inclusion.

Overall, this study provides novel and interesting insights into the requirement for SMN after postnatal mouse development. The work is of high technical quality and the purpose is sound. However, the experimental evidence falls short of supporting the authors' conclusions at critical junctures. Two aspects in particular require significant revision prior to publication.

First, the defects responsible for motor dysfunction, growth impairment and shortened lifespan of ICV injected mice are unclear. Despite an overt phenotype, there is surprisingly little evidence of motor neuron and muscle pathology. Additional experimental work is needed to address the basis of the observed phenotypes. Since morphological and functional disruption of afferent synapses on motor neurons are among the earliest pathological events induced by SMN deficiency in SMA mice (Ling et al PLoS One. 2010:5(11):e15457; Mentis et al. Neuron. 2011:69(3):453-67), dysfunction of neural circuits is a possible mechanism.

Second, the experimental model described here does not accurately phenocopy adult-onset SMA. Type IV SMA patients have moderately reduced SMN expression throughout life rather than a severe SMN depletion in adulthood. Additionally, they present distinctive clinical features including very slow disease progression and normal lifespan, which are not seen in the ASO-induced model characterized by a rapid disease course. The text should be changed to avoid the claim that this model reproduces type IV SMA.

Additional specific points:
- The phenotypic effects of ASO treatment may be caused by excessive depletion of SMN. Additional time points should be analyzed to provide convincing evidence that the onset of motor dysfunction precedes non-specific exacerbation of SMN2 mis-splicing at end-stage.
- Transgenic mice that are homozygous null for the mouse Smn allele and have four copies of the SMN2 gene should not be referred to as "mild-SMA mice" because they do not display SMA-like phenotypes.
- Gems are hardly detectable in Figure 4B. Higher magnification images need to be included.

Referee #3 (Comments on Novelty/Model System):

The authors have previously used ASOs to worsen SMN2 splicing as a way to generate SMA disease like features. The novelty of this study is that they now do it in adult mice. This is of interest to the SMA field as there are not currently models of mild SMA and these may provide important insights into therapeutics that might be targeted specifically to this population.

Referee #3 (General Remarks):

Technically excellent work as has been the norm from this group. The authors have now used ASOs to worsen splicing of SMN2 in adult mice (previously they did it in young mice) and argue that the resulting mice are models of type IV SMA.

I have two concerns about the manuscript:

1) The authors claim to have generated a model of type IV SMA. First I do not support using the terminology used for human disease to mice, so I would stick to "adult onset" SMA for mice. Secondly, even adult onset SMA patients have SMN deficiency for their entire lives (not just during adulthood) and I think the authors need to make clear that their mice are thus a very different genetic situation. Finally, SMA type IV disease pathology is strikingly different from severe forms of SMA in that there is extensive collateral sprouting of motor neuron terminals and evidence of grouped atrophy of muscle (in fact these patients have some of the largest motor units encountered in clinical EMGs). Thus some of the later onset may be due to these compensations. In this study, the authors show no evidence of these pathologies characteristic of the human disease and thus I question somewhat the relevance of model they have generated.
2) The authors shows phenotypes (limited survival and weakness) without a clear pathological explanation-nml MN # and NMJ structure essentially. THis represents a wonderful opportunity to understand the earliest functional changes in motor neurons. The authors have the expertise to do NMJ recordings and I would recommend this as well as an investigation into central synapes.

Additional Author correspondence 13 May 2013

I'm writing in response to your email of March 4th, to thank you for giving us the opportunity to submit a revised manuscript, and to request your advice before we do so.

I should mention that the first author, Dr. Sahashi, left my lab and returned to Japan on March 1, which was rather unfortunate timing. This has made it difficult for us to handle the revision expeditiously, but all the co-authors (at Cold Spring Harbor, San Diego, Los Angeles (but on sabbatical in Taiwan), New York City, Michigan, Stony Brook, and now Nagoya) have now discussed the reviewers' comments and manuscript revision.

We are very pleased with the generally positive comments and constructive suggestions by all three referees. As indicated in the attached point-by-point response, we agree with their comments, and are prepared to revise the text accordingly. With respect to the additional experiments suggested, particularly NMJ electrophysiology and central-synapse histopathology, Referee 3 pointed out that we have the necessary expertise. The reality is that this expertise and the required specialized setup are only available in the Ko lab in Los Angeles; the mice used in our experiments are not kept there, however, so they would have to be treated at Cold Spring Harbor or in San Diego, transported to Los Angeles, and kept out of the vivarium there, because of the quarantine requirements.

Despite the logistical difficulties and substantial cost of such experiments, we acknowledge that in principle they are doable. However, they will take a minimum of two months, if all goes without a glitch, and moreover, we expect the results to be negative; the reason is that the Burghes group already showed, using a tet-responsive promoter, that reduction in SMN levels in 1-month-old mice "resulted in no morphological or electrophysiological abnormalities at the NMJ" (Le et al (2011) Hum Mol Genet 20: 3578-3591). Thus, it is likely that these results will at best end up in the supplement of an already extensive and data-heavy manuscript.

In light of this, we wondered if you might be willing to decide editorially whether a manuscript revised as described in our detailed response, but without the additional experiments, would be acceptable for publication in EMBO Molecular Medicine.

Thank you in advance for your time and advice, and I look forward to your reply.

Referee 1:

This manuscript from Sahashi and colleagues reports on an important set of experiments, detailing the consequences for reducing full-length SMN protein levels in adult SMA mice. The authors have utilised robust antisense oligonucleotide approaches to manipulate splicing of the SMN2 gene in vivo, and they have gone to great lengths to demonstrate the accuracy and repeatability of this approach. The study is very well designed and performed, and represents a huge body of work. The main findings of the study, revealing that disruption of SMN splicing in adult mice can be used to model adult-onset forms of SMA and showing that the adult CNS can tolerate lower levels of SMN protein than in neonatal animals, are well supported by the experimental data. There are, however, a few issues that would need to be addressed before I could recommend publication:

We thank the referee for the very positive overall assessment of our manuscript.

Major points
1) My main concern with the study is the lack of information regarding the effect of the ASOs on control, healthy mice. On page 9, the authors report that "neither ICV nor IP injection of ASO-20-37 affected normal mice with an intact murine Smn gene, ruling out off-target effects". These experiments are very important for the interpretation of the results from experiments in SMA mice and need further clarification. What doses were provided to the control mice and what time-points were examined? How was the health of the mice evaluated? Moreover, given that the SMA mice have liver defects (as previously reported by these authors) is it possible that the SMA mice had a lower tolerance to the ASO and therefore lower doses could elicit toxic effects in vivo? To rule out these possibilities, the authors might want to show that high doses of the ASO (extending beyond those used in the SMA model) have no effects on healthy wild-type mice.

We agree that this is an important point. We injected up to 100 µg ASO-20-37 for ICV or 200 mg/kg/d for IP administration in normal adult mice. We measured body weight for 3-4 months post-surgery and found no growth impairment. In addition, we observed no premature death or overt motor dysfunction during at least 1 year post-surgery. We will mention this in the revised manuscript.

We previously reported liver pathology in severe SMA mice, whereas here we used a much milder SMA model with normal lifespan, whose liver is probably unaffected or much less affected. In addition, we observed mild but significant liver histopathology in the SMA mice with IP injection of a low dose of 50 mg/kg/d ASO-20-37, whereas we saw no pathological changes in normal mice with IP injection of a high dose of 200 mg/kg/d ASO-20-37. These findings rule out the possibility of toxic effects of ASO chemistry in the liver of SMA mice.

We also paid attention to chemistry-related inflammation and thus avoided administration of excessive ASO doses, to be able to demonstrate that the disease was specifically elicited through *SMN2* splicing inhibition.

2) The authors show in Fig 2C that IP delivery of their ASO had limited effects on splicing in muscle (and to a lesser extent heart). Given that both muscle and heart are thought to be directly affected in SMA, might these results have implications for the severity of the resulting phenotype observed in the SMA mice? For example, the lack of muscle pathology that the authors observe (page 10) could potentially be explained by the lack of changes in full-length protein expression (e.g. muscle pathology might be expected to occur if the ASO successfully targeted muscle), rather than indicating that muscle pathology is only a late event in the disease (as the authors propose).

We acknowledge that this is a plausible alternative explanation, and we will include it in a revised discussion. The ASO has an effect on *SMN2* splicing in muscle, but it is certainly possible that our approach does not precisely recapitulate in a quantitative manner the extent of defective splicing (and hence the precise SMN protein thresholds) present in each tissue in SMA patients or classical mouse models.

3) Given the growing body of evidence for the involvement of peripheral tissues in SMA other than

the liver (as previously shown by these authors), such as heart, lungs, pancreas, GI tract, bone etc (recently reviewed in PMID: 23228902), did the authors observe any other systemic pathology in the ASO-treated SMA mice? Did they check for splicing effects in any other organs or tissues in the mice treated with ASO via IP delivery?

Considering the biodistribution of IP-injected ASO and our published work, we initially focused on analysis of peripheral organs, such as liver, muscle, and heart. We are aware of the potential relevance of other peripheral tissues, and we plan to extend the analysis to such tissues in the future, but we feel that this is beyond the scope of the present manuscript, which is already very extensive.

Minor comments:
1) On page 6 (second last sentence), the authors refer to data from PS7 and PS14 in Fig 1, but only the former is shown.

We will correct the description in the figure; we performed a dose-response study of ASO-20-37's effects at PS7, and checked that the control ASO had no effect on splicing at either PS7 or PS14.

2) I found the discussion to be a bit too long (just a personal opinion, I acknowledge!). As a result, some of the main messages got rather lost by the end. It may benefit from a bit of judicious pruning.

We will streamline the Discussion section.

3) It is unclear what the graphs are actually showing in Fig 2A and Fig 2B. The figure legends need to be amended accordingly.

Both bar graphs indicate the percentage of full-length transcript. We will make this clear in the revised manuscript.

4) It would be helpful for N numbers and statistical tests to be included in all the figure legends.

We indicated in Methods that we used three biological replicates for RT-PCR analyses and the statistical tests we used. We will also provide similar information in the figure legends in the revised manuscript.

Referee #2:

This manuscript describes the phenotypic effects of lowering SMN expression in adult mice using antisense oligonucleotides (ASOs) that target SMN2 exon 7 splicing. The spatial requirement for SMN is investigated through behavioural and histopathological analysis following ASO injection either in the CNS or in the periphery. Although low SMN levels in either compartment lead to weight loss and lethality, the mechanisms appear remarkably distinct. SMN depletion in the CNS affects motor function, while low SMN levels in the periphery elicit liver and heart pathology but have no effects on the neuromuscular system. As expected, these phenotypes are corrected by injection of a therapeutic ASO that restores SMN expression by promoting exon 7 inclusion.

Overall, this study provides novel and interesting insights into the requirement for SMN after postnatal mouse development. The work is of high technical quality and the purpose is sound. However, the experimental evidence falls short of supporting the authors' conclusions at critical junctures. Two aspects in particular require significant revision prior to publication.

We thank the referee for the positive overall comments, and we address the two major aspects below.

First, the defects responsible for motor dysfunction, growth impairment and shortened lifespan of ICV injected mice are unclear. Despite an overt phenotype, there is surprisingly little evidence of motor neuron and muscle pathology. Additional experimental work is needed to address the basis of the observed phenotypes. Since morphological and functional disruption of afferent synapses on motor neurons are among the earliest pathological events induced by SMN deficiency in SMA mice (Ling et al PLoS One. 2010:5(11):e15457; Mentis et al. Neuron. 2011:69(3):453-67), dysfunction of

neural circuits is a possible mechanism.

Although there was no evidence of motor-neuron loss in PS30 lumbar spinal cord after ICV injection of 100 µg ASO-20-37, we did observe shrinkage of motor neurons (Fig. 4B), indicative of neuronal degeneration. However, to further address the underlying mechanism of motor dysfunction, we agree that it may prove informative to analyse central synapse histology, to look for evidence of abnormal neural circuits. But given the time, cost and expertise required for such experiments, we would prefer to leave such experiments for future follow-up work.

Second, the experimental model described here does not accurately phenocopy adult-onset SMA. Type IV SMA patients have moderately reduced SMN expression throughout life rather than a severe SMN depletion in adulthood. Additionally, they present distinctive clinical features including very slow disease progression and normal lifespan, which are not seen in the ASO-induced model characterized by a rapid disease course. The text should be changed to avoid the claim that this model reproduces type IV SMA.

We agree that we should be more careful in describing the extent to which our approach phenocopies type IV SMA. The mice we used have basal SMN levels that are lower than those in normal mice, and we decrease SMN further by injecting the ASO in adult mice; the extent of this decrease is dose-dependent. Except for the tail and ear necrosis, our mice display an asymptomatic phase, with moderately reduced SMN levels, before disease onset at the adult stage. Compared with type IV SMA, the disease progression after onset is more rapid and without chronic compensation, but some characteristic phenotypes are clearly manifested. In any case, we will provide a more balanced discussion in comparing and contrasting the phenocopy model from actual type IV SMA.

Additional specific points:
The phenotypic effects of ASO treatment may be caused by excessive depletion of SMN. Additional time points should be analysed to provide convincing evidence that the onset of motor dysfunction precedes non-specific exacerbation of SMN2 mis-splicing at end-stage.

Mice treated with ICV injection of 100 µg of ASO-20-37 exhibited no decline in rotarod tasks performed at PS20, although we did not determine when exacerbation of SMN mis-splicing exactly begins. Despite marked inhibition of *SMN2* splicing as early as PS7, these ICV-injected mice displayed gait disturbance in addition to locomotor decline only at the late disease stages. In contrast, no overt gait disturbance was observed in IP-injected mice, even during end-stage disease, suggesting that motor dysfunction is not merely secondary to end-stage disease conditions. We will discuss this more explicitly in the revised manuscript.

Transgenic mice that are homozygous null for the mouse Smn allele and have four copies of the SMN2 gene should not be referred to as "mild-SMA mice" because they do not display SMA-like phenotypes.

We agree; we will use more precise terminology in the revised manuscript.

Gems are hardly detectable in Figure 4B. Higher magnification images need to be included.

We will include higher-magnification images in the revised Fig 4B.

Referee #3:

The authors have previously used ASOs to worsen SMN2 splicing as a way to generate SMA disease like features. The novelty of this study is that they now do it in adult mice. This is of interest to the SMA field as there are not currently models of mild SMA and these may provide important insights into therapeutics that might be targeted specifically to this population.

Technically excellent work as has been the norm from this group. The authors have now used ASOs to worsen splicing of SMN2 in adult mice (previously they did it in young mice) and argue that the resulting mice are models of type IV SMA.

We thank the referee for the overall positive assessment.

I have two concerns about the manuscript:

1) The authors claim to have generated a model of type IV SMA. First I do not support using the terminology used for human disease to mice, so I would stick to "adult onset" SMA for mice. Secondly, even adult onset SMA patients have SMN deficiency for their entire lives (not just during adulthood) and I think the authors need to make clear that their mice are thus a very different genetic situation. Finally, SMA type IV disease pathology is strikingly different from severe forms of SMA in that there is extensive collateral sprouting of motor neuron terminals and evidence of grouped atrophy of muscle (in fact these patients have some of the largest motor units encountered in clinical EMGs). Thus some of the later onset may be due to these compensations. In this study, the authors show no evidence of these pathologies characteristic of the human disease and thus I question somewhat the relevance of model they have generated.

We appreciate these comments, which are related to those of Reviewer #2. We agree that SMN levels are not constantly low in our phenocopy model, which is distinct from the situation in type IV SMA patients, and also agree that we should refer to our mice as an adult-onset SMA model. Note, however, that SMN levels are below normal before ASO treatment, due to the genotype, and then they are further reduced by ASO treatment, in a dose-dependent manner.

The relatively rapid disease course may prevent chronic compensatory changes in our phenocopy model, which thus may not account for the delayed-onset motor phenotype. However, compared with our previous finding that P1 ICV injection causes progressive paralysis, beginning after ~2 weeks, the present study suggests that the late-onset motor phenotype reflects important differences between development and post-development.

We will discuss the above similarities and differences in the revised manuscript.

2) The authors show phenotypes (limited survival and weakness) without a clear pathological explanation-normal MN # and NMJ structure essentially. This represents a wonderful opportunity to understand the earliest functional changes in motor neurons. The authors have the expertise to do NMJ recordings and I would recommend this as well as an investigation into central synapse.

Although we did observe morphological changes in motor neurons and NMJs in ICV-injected mice, we agree that performing electrophysiological NMJ analysis to further clarify the mechanism of motor dysfunction may prove informative. However, the expertise to do such work is available in the Ko lab, but not in the Krainer lab or at Isis; treating and then moving mice to a distant location for analysis is a logistical challenge. We would prefer to do such experiments in future follow-up work.

Additional Editorial Correspondence 15 May 2013

Thank you for your letter. I have now discussed it with my colleagues and with the Reviewer.

The main concern of this Reviewer was the lack of an explanation for why the late adult onset SMA mice show weakness and reduced survival. The assumption that this phenotype relates primarily to abnormalities of the neuromuscular system and thus is relevant to human SMA was not thoroughly demonstrated, as not much evidence of a histological abnormality of the motor unit was provided.

This is the reason why s/he asked for more functional studies. For instance, a possibility is that weakness and reduced survival is due to some other organ system abnormality such as cardiac disease, which has been observed in other SMA mouse models. If such case, the usefulness of these mice in modeling late-onset SMA, would be called into question. Indeed, both Reviewer 1 (especially) and 2 did raise similar issues.

So at the end of the story, it is not a matter of performing or not specific experiments, but of providing persuasive evidence supporting the relevance of the model for late-onset SMA. It is thus

important that you address these concerns directly and convincingly.

I hope this is useful for your continued effort. If you should require more time, just let us know.

1st Revision received 06 July 2013

Referee #1:

This manuscript from Sahashi and colleagues reports on an important set of experiments, detailing the consequences for reducing full-length SMN protein levels in adult SMA mice. The authors have utilised robust antisense oligonucleotide approaches to manipulate splicing of the SMN2 gene in vivo, and they have gone to great lengths to demonstrate the accuracy and repeatability of this approach. The study is very well designed and performed, and represents a huge body of work. The main findings of the study, revealing that disruption of SMN splicing in adult mice can be used to model adult-onset forms of SMA and showing that the adult CNS can tolerate lower levels of SMN protein than in neonatal animals, are well supported by the experimental data. There are, however, a few issues that would need to be addressed before I could recommend publication:

We thank the referee for the very positive overall assessment of our manuscript.

Major points
1) My main concern with the study is the lack of information regarding the effect of the ASOs on control, healthy mice. On page 9, the authors report that "neither ICV nor IP injection of ASO-20-37 affected normal mice with an intact murine Smn gene, ruling out off-target effects". These experiments are very important for the interpretation of the results from experiments in SMA mice and need further clarification. What doses were provided to the control mice and what time-points were examined? How was the health of the mice evaluated? Moreover, given that the SMA mice have liver defects (as previously reported by these authors) is it possible that the SMA mice had a lower tolerance to the ASO and therefore lower doses could elicit toxic effects in vivo? To rule out these possibilities, the authors might want to show that high doses of the ASO (extending beyond those used in the SMA model) have no effects on healthy wild-type mice.

We agree that this is an important point. We injected up to 100 µg ASO-20-37 for ICV or 200 mg/kg/d for IP administration in normal adult mice. We measured body weight for 3-4 months post-surgery and found no growth impairment. In addition, we observed no premature death or overt motor dysfunction during at least 1 year post-surgery. We mention this in the revised manuscript (Page 9, line 9).

We previously reported liver pathology in severe SMA mice, whereas here we used a much milder SMA model with normal lifespan, whose liver is probably unaffected or much less affected. In addition, we observed mild but significant liver histopathology even in the SMA mice that received low-dose IP injection of 50 mg/kg/d ASO-20-37, whereas we saw no pathological changes in normal mice that received high-dose IP injection of 200 mg/kg/d ASO-20-37. These findings rule out the possibility of toxic effects of ASO chemistry in the liver of SMA mice. We mention this in the revised manuscript (Page 13, line 12).

We also avoided chemistry-related inflammation due to administration of excessive ASO doses, to be able to demonstrate that the disease was specifically elicited through *SMN2* splicing inhibition.

2) The authors show in Fig 2C that IP delivery of their ASO had limited effects on splicing in muscle (and to a lesser extent heart). Given that both muscle and heart are thought to be directly affected in SMA, might these results have implications for the severity of the resulting phenotype observed in the SMA mice? For example, the lack of muscle pathology that the authors observe (page 10) could potentially be explained by the lack of changes in full-length protein expression (e.g. muscle pathology might be expected to occur if the ASO successfully targeted muscle), rather than indicating that muscle pathology is only a late event in the disease (as the authors propose).

We acknowledge that this is a plausible alternative explanation, and we included it in the revised

manuscript (Page 10, line 22; Page 16, line 21). The ASO has an effect on *SMN2* splicing in muscle and heart, but it is certainly possible that our approach does not precisely recapitulate in a quantitative manner the extent of defective splicing (and hence the precise SMN protein thresholds) present in each tissue in SMA patients or classical mouse models.

3) Given the growing body of evidence for the involvement of peripheral tissues in SMA other than the liver (as previously shown by these authors), such as heart, lungs, pancreas, GI tract, bone etc (recently reviewed in PMID: 23228902), did the authors observe any other systemic pathology in the ASO-treated SMA mice? Did they check for splicing effects in any other organs or tissues in the mice treated with ASO via IP delivery?

Considering the biodistribution of IP-injected ASO and our published studies, we initially focused on analysis of peripheral organs, such as liver, muscle, and heart. We are aware of the potential relevance of other peripheral tissues, and we plan to extend the analysis to such tissues in the future, but we feel that this is beyond the scope of the present manuscript, which is already very extensive.

Minor comments:
1) On page 6 (second last sentence), the authors refer to data from PS7 and PS14 in Fig 1, but only the former is shown.

We corrected the description in the revised manuscript (Page 6, line 25); we performed a dose-response study of ASO-20-37's effects at PS7, and checked that the control ASO had no effect on splicing at either PS7 or PS14.

2) I found the discussion to be a bit too long (just a personal opinion, I acknowledge!). As a result, some of the main messages got rather lost by the end. It may benefit from a bit of judicious pruning.

We streamlined the Discussion section.

3) It is unclear what the graphs are actually showing in Fig 2A and Fig 2B. The figure legends need to be amended accordingly.

Both bar graphs indicate the percentage of full-length transcript. We made this clear in the figure legends in the revised manuscript.

4) It would be helpful for N numbers and statistical tests to be included in all the figure legends.

We indicated in Methods that we used three biological replicates for RT-PCR analyses and the statistical tests we used. We now provide similar information in the figure legends in the revised manuscript.

Referee #2:

This manuscript describes the phenotypic effects of lowering SMN expression in adult mice using antisense oligonucleotides (ASOs) that target SMN2 exon 7 splicing. The spatial requirement for SMN is investigated through behavioral and histopathological analysis following ASO injection either in the CNS or in the periphery. Although low SMN levels in either compartment lead to weight loss and lethality, the mechanisms appear remarkably distinct. SMN depletion in the CNS affects motor function, while low SMN levels in the periphery elicit liver and heart pathology but have no effects on the neuromuscular system. As expected, these phenotypes are corrected by injection of a therapeutic ASO that restores SMN expression by promoting exon 7 inclusion.

Overall, this study provides novel and interesting insights into the requirement for SMN after postnatal mouse development. The work is of high technical quality and the purpose is sound. However, the experimental evidence falls short of supporting the authors' conclusions at critical junctures. Two aspects in particular require significant revision prior to publication.

We thank the referee for the positive overall comments, and we address the two major aspects below.

First, the defects responsible for motor dysfunction, growth impairment and shortened lifespan of ICV injected mice are unclear. Despite an overt phenotype, there is surprisingly little evidence of motor neuron and muscle pathology. Additional experimental work is needed to address the basis of the observed phenotypes. Since morphological and functional disruption of afferent synapses on motor neurons are among the earliest pathological events induced by SMN deficiency in SMA mice (Ling et al PLoS One. 2010:5(11):e15457; Mentis et al. Neuron. 2011:69(3):453-67), dysfunction of neural circuits is a possible mechanism.

We actually did observe clear motor-neuron, NMJ, and muscle phenotypes in ICV-injected mice, and we now state this more clearly and explicitly in the revised manuscript (Pages 9-12, 17). In particular: (1) the number of gems in spinal motor neurons was markedly decreased by 75-85% after ICV injection of 100 µg or 25 µg ASO, which is expected to cause neuronal dysfunction; (2) a significant 45% loss, as well as shrinkage of motor neurons (Fig. 4B), indicative of neuronal degeneration, were observed in mice ICV-injected with 25 µg ASO—a dose compatible with sufficiently long survival to develop motor-neuron degeneration; (3) abnormal NMJ phenotypes, such as varicosities in the nerve terminals and endplate fragmentation, were seen in mice ICV-injected with 25 µg ASO, and these defects were even more prominent in mice treated with combined ICV and IP ASO injections. These types of structural changes in NMJs are characteristic of aged, damaged, or diseased muscles, in which NMJs undergo extensive remodeling (Li et al. 2011; Li and Thompson 2011). The fact that we observe these changes in response to post-developmental SMN deficiency suggests, for the first time, that SMN function is required for maintaining the NMJ structure in adulthood.

However, to further address the origin of motor deficits, we agree that it should be informative to analyze the central synapses between proprioceptive afferents and motor neurons, early defects in which potentially affect motor-neuron excitability and motor circuitry in SMA (Ling et al. 2010; Mentis et al. 2011). We have now examined these synapses by immunostaining in mice ICV-injected with 100 µg ASO—for which we did not observe morphological defects in motor neurons or NMJs during their short lifespan—and we indeed found a significant 25% loss of synaptic boutons.

Taken together, these results indicate that ASO-20-37 elicits SMA-like motor-unit pathologies, as well as motor dysfunction, in our phenocopy model. We added the new data about the synaptic defect (Fig 4F), and the text in the revised Results and Discussion was changed to highlight this pathological phenotype (Page 11, beginning on line 23; Page 17, beginning on line 11).

Despite the above-mentioned structural changes in NMJs, our results show that NMJs remained fully innervated in mice treated with ICV injection or combined ICV and IP injections of ASO-20-37, or in mice additionally treated with therapeutic ASO. Although we did not further investigate functional aspects of NMJs, Le et al. (2011) provided insights into the neuromuscular function of mice with SMN deficiency in adulthood. They generated a transgenic SMA mouse line that over-expresses full-length SMN upon induction. Early postnatal induction prolonged the survival of these SMA mice. However, when the induction was stopped after 1 month, most mice became weak and died one month later, without showing any abnormal NMJ electrophysiology. This study suggests that SMN depletion in adulthood does not affect NMJs in such a short term. Based on their finding, it is unlikely that our mice ICV-injected with 100 µg ASO, which die within one month, will show a disruption in neuromuscular functions.

We believe that motor-neuron pathology primarily causes weakness, including muscle weakness, in ICV-injected mice. Respiratory distress due to denervation of bulbar or respiratory muscles—common in severe SMA—should also be taken into account in these mice, because *SMN2* splicing is especially inhibited in the CNS. The resulting dysphagia and/or respiratory distress are expected to result in reduced food intake, and, in turn, to cause nutritional deficiency, exacerbating *SMN2* mis-splicing and SMA severity. This could partly explain the observed growth impairment, as well as shortened lifespan.

Second, the experimental model described here does not accurately phenocopy adult-onset SMA. Type IV SMA patients have moderately reduced SMN expression throughout life rather than a severe SMN depletion in adulthood. Additionally, they present distinctive clinical features including

very slow disease progression and normal lifespan, which are not seen in the ASO-induced model characterized by a rapid disease course. The text should be changed to avoid the claim that this model reproduces type IV SMA.

We agree that we should be more careful in describing the extent to which our approach phenocopies type IV SMA. Except for the tail and ear necrosis, the transgenic mice we used shares with type IV SMA an asymptomatic phase, with moderately reduced SMN levels, before disease onset at the adult stage. Although our mice have basal SMN levels that are lower than those in normal mice, we decrease SMN further, in a tissue-specific manner, by injecting ASO-20-37 in adult mice; the extent of this decrease is dose-dependent. This situation is distinct from that in type IV SMA, and disease progression after onset is more rapid and without chronic compensation. However, some characteristic phenotypes, SMN's temporal and spatial roles in SMA pathogenesis, and a broad therapeutic time window for adult-onset SMA are clearly revealed. We provided a more balanced discussion in comparing and contrasting the phenocopy model with actual type IV SMA (Page 20, line 22).

Additional specific points:
The phenotypic effects of ASO treatment may be caused by excessive depletion of SMN. Additional time points should be analyzed to provide convincing evidence that the onset of motor dysfunction precedes non-specific exacerbation of SMN2 mis-splicing at end-stage.

Mice treated with ICV injection of 100 µg of ASO-20-37 exhibited no decline in rotarod tasks performed at PS20, although we did not determine when exacerbation of *SMN2* mis-splicing exactly begins. Despite marked inhibition of *SMN2* splicing as early as PS7, these ICV-injected mice displayed gait disturbance in addition to locomotor decline only at the late disease stages, accompanied by SMA-like pathologies in motor neurons, NMJs, and central synapses. In contrast, no overt gait disturbance was observed in IP-injected mice, even during end-stage disease, suggesting that motor dysfunction is not merely secondary to end-stage disease conditions. We discussed this explicitly in the revised manuscript (Page 16, line 14).

Transgenic mice that are homozygous null for the mouse Smn allele and have four copies of the SMN2 gene should not be referred to as "mild-SMA mice" because they do not display SMA-like phenotypes.

We agree; we used more appropriate terminology in the revised manuscript.

Gems are hardly detectable in Figure 4B. Higher magnification images need to be included.

We included higher-magnification images in the revised Fig 4A.

Referee #3:

The authors have previously used ASOs to worsen SMN2 splicing as a way to generate SMA disease like features. The novelty of this study is that they now do it in adult mice. This is of interest to the SMA field as there are not currently models of mild SMA and these may provide important insights into therapeutics that might be targeted specifically to this population.

Technically excellent work as has been the norm from this group. The authors have now used ASOs to worsen splicing of SMN2 in adult mice (previously they did it in young mice) and argue that the resulting mice are models of type IV SMA.

We thank the referee for the overall positive assessment.

I have two concerns about the manuscript:

1) The authors claim to have generated a model of type IV SMA. First I do not support using the terminology used for human disease to mice, so I would stick to "adult onset" SMA for mice. Secondly, even adult onset SMA patients have SMN deficiency for their entire lives (not just during adulthood) and I think the authors need to make clear that their mice are thus a very different

genetic situation. Finally, SMA type IV disease pathology is strikingly different from severe forms of SMA in that there is extensive collateral sprouting of motor neuron terminals and evidence of grouped atrophy of muscle (in fact these patients have some of the largest motor units encountered in clinical EMGs). Thus some of the later onset may be due to these compensations. In this study, the authors show no evidence of these pathologies characteristic of the human disease and thus I question somewhat the relevance of model they have generated.

We appreciate these comments, which are related to those of Reviewer #2. We agree that SMN levels are not constantly low in our phenocopy model, which is distinct from the situation in type IV SMA patients, and also agree that we should refer to our mice as an adult-onset SMA model. Note, however, that SMN levels are below normal before ASO treatment in an asymptomatic phase, due to the genotype, and then they are further reduced by ASO treatment, in a dose-dependent manner.

The relatively rapid disease course may prevent chronic compensatory changes in our phenocopy model, which thus may not account for the delayed-onset motor phenotype. However, compared with our previous finding that P1 ICV injection causes progressive paralysis, beginning after ~2 weeks, the present study suggests that, though accompanied by motor-unit pathologies, the late-onset motor phenotype is due to the mature motor units tolerating SMN deficiency, and thus the phenotype reflects important differences between development and post-development.

We discussed the above similarities and differences in the revised manuscript (Page 16, beginning on line 12; Page 17, beginning on line 14).

2) The authors show phenotypes (limited survival and weakness) without a clear pathological explanation-normal MN # and NMJ structure essentially. This represents a wonderful opportunity to understand the earliest functional changes in motor neurons. The authors have the expertise to do NMJ recordings and I would recommend this as well as an investigation into central synapse.

We did observe a marked loss of nuclear gems in motor neurons, as well as morphological changes in motor neurons and NMJs in ICV-injected mice. However, in response to this comment and a similar one by Reviewer #2, we went ahead and analyzed central synapses. In the revised manuscript, we show the new data demonstrating a significant loss of synaptic boutons, further documenting the SMA-like motor phenotypes (Page 11, starting on line 23; Page 17, starting on line 11), as discussed above in our response to Reviewer #2's comment.

We agree that performing electrophysiological NMJ analysis to further clarify the mechanism of motor dysfunction may prove informative. However, the expertise to do such work is available in the Ko lab, but not in the Krainer lab or at Isis; treating and then moving these weak mice to a distant location for analysis is a logistical challenge. We would prefer to do such experiments in future follow-up work. In addition, as discussed in our response to Reviewer #2's comment, defects in neuromuscular transmission are unlikely in ICV-injected mice.

2nd Editorial Decision 23 July 2013

Thank you for the submission of your revised manuscript to EMBO Molecular Medicine. We have now heard back from the three Reviewers whom we asked to evaluate your manuscript.

You will see that while the Reviewers are supportive of you work, a few issues remain to be acted upon (mostly relative to the new data) before we can accept your manuscript for publication.

Please fully address the Reviewer 2 and 3's remaining concerns as quickly as possible and in any case within two weeks. Provided these issues are fully addressed, the final decision will be made at the Editorial level.

I look forward to receiving your re-revised manuscript as soon as possible

***** Reviewer's comments *****

Referee #1 (General Remarks):

The authors have addressed all my concerns and revised the manuscript accordingly.

Referee #2 (General Remarks):

The authors have satisfactorily addressed most of my comments in their revision. In particular, the new data on the effects of SMN deficiency on proprioceptive synapses on motor neurons significantly strengthens this study. Deafferentation of motor neurons appears as the only morphological abnormality in the sensory-motor circuit of mice injected with the highest dose of ASO (100 g). Although functional analysis by electrophysiology is not performed, this finding strongly implicates deficits in the central synapses of motor neurons as determinants of the phenotypes induced by SMN deficiency in the adult CNS.

I would recommend publication after the following issues are addressed.

1) There appears to be ChAT colocalization with VGluT1 synapses (Figure 4F), questioning the specificity of the staining. Images of each individual channel must be shown to demonstrate that this is not the case.

2) C boutons on motor neurons seem much less numerous in ASO-treated than in control animals. Quantification of the number of C boutons should be included in Figure 4F. If they are indeed reduced, it would point to a more general requirement of SMN for the maintenance of central synapses on adult motor neurons than just proprioceptive ones.

3) Varicosities in the nerve terminals and fragmentation of motor endplates are taken as evidence of motor neuron pathology in mice injected with 25 g ASO (Figure 4D). Since these defects appear modest if not negligible, the manuscript should clearly indicate that they are at least statistically significant.

4) The use of "type-I-like" and "type-II-like" when referring to SMA mice should be avoided (pages 3, 4 and 6).

Referee #3 (General Remarks):

The authors have addressed my concerns. Two minor recomendations regarding wording:
1) In the abstract, the word "phenocopies" in the sentence "..ICV ASO injection in adult SMN2 transgenic mice phenocopies adult onset SMA.." is a little strong. The muscle pathology is clearly distinct in these two situations. How about "resembles" instead?
2)First sentence of introduction...it is still debated to some degreee how "progressive" the weakness and atrophy is in the SMA patients after the early period of the disease. Suggest removing this word.

2nd Revision received 06 August 2013

Referee #1:

The authors have addressed all my concerns and revised the manuscript accordingly.

No further changes were requested by this referee.

Referee #2:

The authors have satisfactorily addressed most of my comments in their revision. In particular, the new data on the effects of SMN deficiency on proprioceptive synapses on motor neurons significantly strengthens this study. Deafferentation of motor neurons appears as the only morphological abnormality in the sensory-motor circuit of mice injected with the highest dose of ASO (100 µg). Although functional analysis by electrophysiology is not performed, this finding strongly implicates deficits in the central synapses of motor neurons as determinants of the phenotypes induced by SMN deficiency in the adult CNS.

I would recommend publication after the following issues are addressed.

1) There appears to be ChAT colocalization with VGluT1 synapses (Figure 4F), questioning the specificity of the staining. Images of each individual channel must be shown to demonstrate that this is not the case.

The apparent overlap between ChAT and VGLUT1 signals was due to green-to-red channel bleed-through, but did not affect the quantification of the VGLUT1 green signal. We solved this technical problem by using different secondary antibodies. We also included separate ChAT and VGLUT1 images, in addition to the merged ones, in the revised Fig 4F.

2) C boutons on motor neurons seem much less numerous in ASO-treated than in control animals. Quantification of the number of C boutons should be included in Figure 4F. If they are indeed reduced, it would point to a more general requirement of SMN for the maintenance of central synapses on adult motor neurons than just proprioceptive ones.

We agree that these data provide further evidence of central-synapse pathology. We counted the number of C-boutons per motor neuron, and found a significant 35% reduction in the mice treated with 100 µg exon-skipping ASO. We included the quantitation in the revised manuscript (Page 19, line 9; Page 17, line 13).

3) Varicosities in the nerve terminals and fragmentation of motor endplates are taken as evidence of motor neuron pathology in mice injected with 25 µg ASO (Figure 4D). Since these defects appear modest if not negligible, the manuscript should clearly indicate that they are at least statistically significant.

We observed more fragmented endplates, and varicosities in the nerve terminals in mice treated with 25 µg ASO ICV, but not with 100 µg ASO; due to the small sample size, the data did not reach statistical significance. However, combined ICV and IP injection of ASO-20-37 elicited a significantly higher incidence of both defects, as well as a reduction in mature-pretzel forms of AChR. These results suggest that adult-onset SMN deficiency can affect NMJ maintenance. We discussed this in the revised manuscript (Page 11, lines 8 and 18).

4) The use of "type-I-like" and "type-II-like" when referring to SMA mice should be avoided (pages 3, 4 and 6).

We no longer use this terminology (Page 3, line 14; Page 4, line 10; Page 6, line 14).

Referee #3 (General Remarks):

The authors have addressed my concerns. Two minor recommendations regarding wording:

1) In the abstract, the word "phenocopies" in the sentence "..ICV ASO injection in adult SMN2 transgenic mice phenocopies adult onset SMA.." is a little strong. The muscle pathology is clearly distinct in these two situations. How about "resembles" instead?

We used the term “phenocopy” for neonate mice in our original TSUNAMI manuscript (Sahashi et al. 2012) and we would prefer to use consistent terminology for the present work with adult mice. We do not wish to imply that our ASO-induced model is an exact phenocopy of SMA, but this is made clear in the Discussion. We have modified the sentence to read “… phenocopies key aspects of adult-onset SMA …” in the revised abstract.

2) First sentence of introduction...it is still debated to some degree how "progressive" the weakness and atrophy is in the SMA patients after the early period of the disease. Suggest removing this word.

We deleted the word “progressive” in line 2 of Page 3.
