# Supplementary material for: Pathological impact of SMN2 mis-splicing in adult SMA mice
Source: EMBO Mol Med. 2013 Sep 9;5(10):1586–601. doi: 10.1002/emmm.201302567 (PMC3799581; doi:10.1002/emmm.201302567)
Supplement: Supplementary file 2 [file emmm0005-1586-SD2.pdf]

Supporting Information

Pathological impact of *SMN2* mis-splicing in adult SMA mice

Kentaro Sahashi, Yimin Hua, Karen K. Y. Ling, J. Erby Wilkinson, Tomoki Nomakuchi, Frank Rigo, Gene Hung, David Xu, Ya-Ping Jiang, Richard Z. Lin, Chien-Ping Ko, C. Frank Bennett, and Adrian R. Krainer

Supporting Information Figures

Figure S1. Splicing effect of control ASO, therapeutic ASO-10-27, or ASO-20-37

Figure S2. ASO uptake in tissues 7 or 30 days after ICV injection of 100 µg ASO-20-37

Figure S3. Phenotypic effect of ASO-20-37

Figure S4. Muscle histology

Figure S5. Amelioration by systemically administered therapeutic ASO

Supporting Information Tables

Table S1. Sequences of MOE ASOs used in this study

Table S2. Echocardiographic measurements before and 25 days after IP injection of control ASO or ASO-20-37

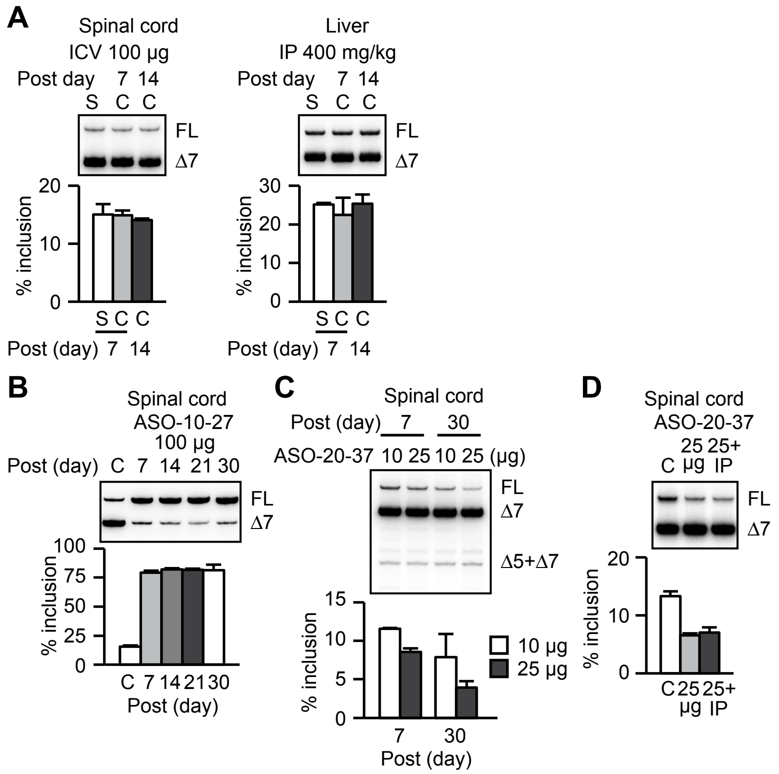

Figure S1. Splicing effect of control ASO, therapeutic ASO-10-27, or ASO-20-37. (**A**) ICV injection of 100 µg or IP injection of 200 mg/kg/d control ASO did not affect *SMN2* splicing in spinal cord or liver, respectively, analyzed at PS7 or PS14 (n = 3). (**B**) Time-course analysis of the effect of ICV injection of 100 µg ASO-10-27 on *SMN2* splicing in spinal cord (n = 3). (**C**) Compared with PS7, 10 µg or 25 µg ASO-20-37 further inhibited exon 7 inclusion at PS30 in a dose-dependent manner (n = 3). 25 µg ASO has a stronger effect than 10 µg. (**D**) Similar splicing effect in PS7 spinal cord between ICV injection of 25 µg ASO-20-37 with or without additional IP injection of 200 mg/kg/d ASO-20-37 (n = 3). FL, Δ7, and Δ5+Δ7: full-length, exon-7-skipped transcripts, and mRNA lacking both exon 5 and exon 7, respectively. S: 5 µl saline, C: 100 µg control ASO; 25+IP: combination of ICV injection of 25 µg and IP injection of 200 mg/kg/d ASO-20-37.!

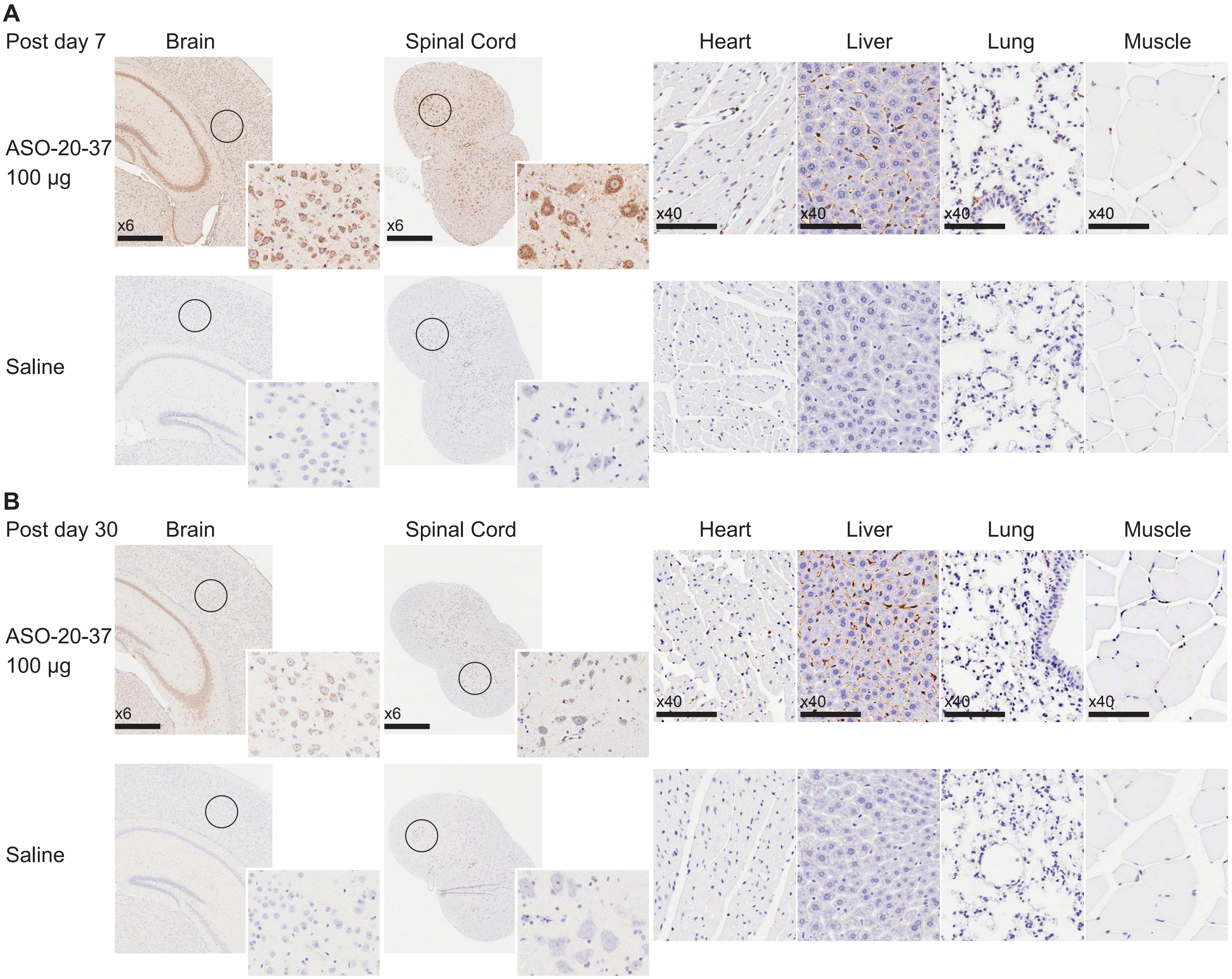

Figure S2. ASO uptake in tissues 7 days (**A**) or 30 days (**B**) after ICV injection of 100 µg ASO-20-37 (IHC). ASO is broadly detected in CNS cells, and mainly in liver sinusoids. ASO is only weakly detectable in heart, lung, and quadriceps muscle. Compared with PS7, PS30 uptake declines in the CNS. The brain and spinal cord regions indicated by open circles are also displayed at higher magnification in the insets. Scale bar: 1,000 and 200 µm for 6× and 40× magnification. Saline: ICV injection of 5 µl saline.

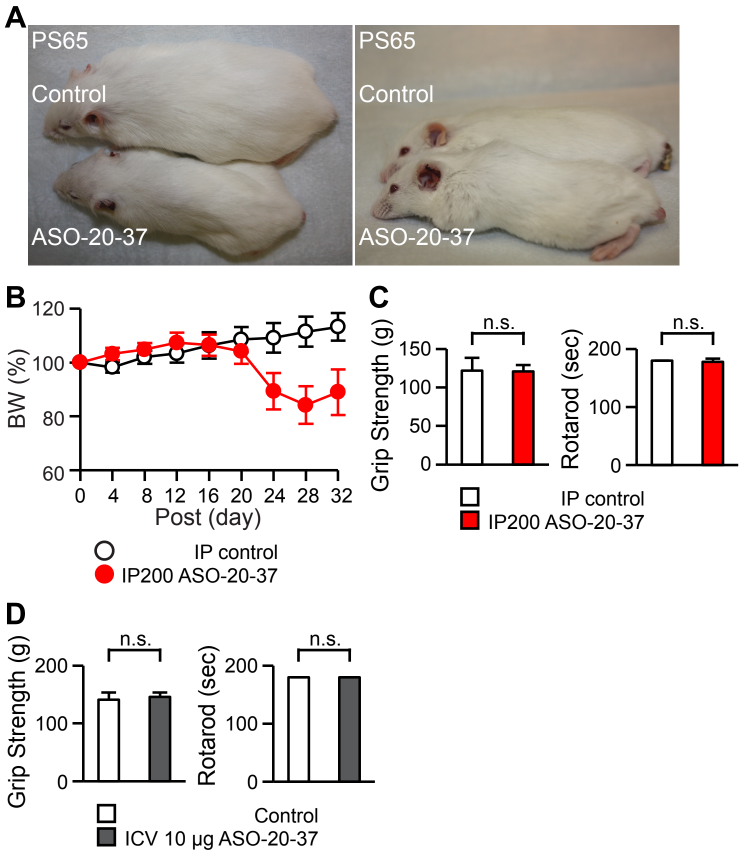

Figure S3. Phenotypic effect of ASO-20-37. (**A**) Mice that receive ICV injection of 25 μg ASO-20-37 are smaller and show kyphosis. (**B**) Mice begin to lose body weight around 20 days after IP injection of 200 mg/kg/d ASO-20-37. Control mice (n = 10) and mice injected with ASO-20-37 (n = 12) were analyzed. (**C**) IP injection of ASO-20-37 had no effect on motor function, analyzed by grip strength (n = 7. n.s., $P = 0.9122$) and rotarod task (n = 7. n.s., $P = 0.3599$) measured at PS21. (**D**) ICV injection of 10 μg ASO-20-37 had no effect on motor function, analyzed by grip strength (n = 3. n.s., $P = 0.6601$) and rotarod task (n = 3. n.s., $P = 0.4227$) measured at PS120. Control: ICV injection of 100 μg control ASO; IP control: IP injection of 200 mg/kg/d control ASO. We analyzed data using two-tailed *t*-tests.

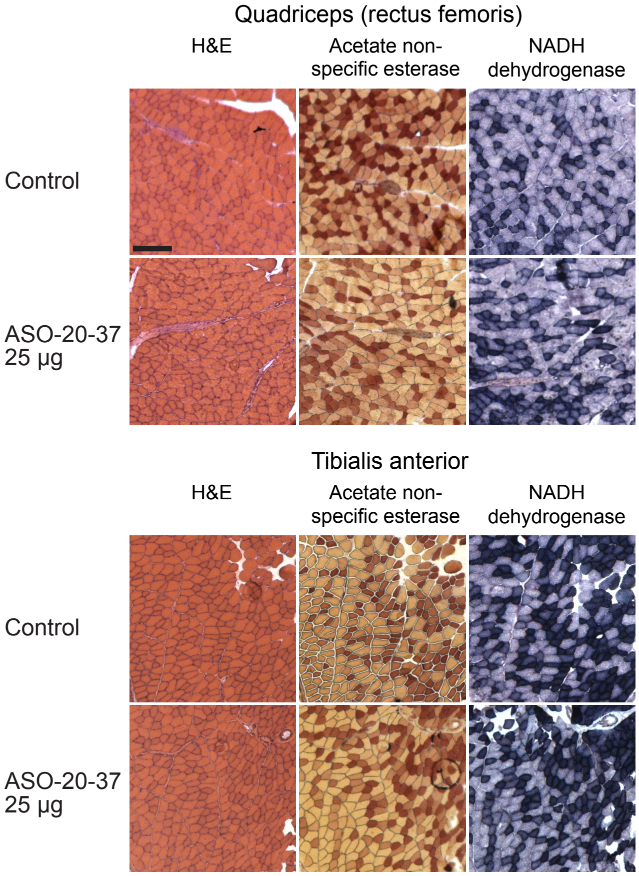

Figure S4. Muscle histology. No overt muscle pathology in quadriceps (top) or tibialis anterior (bottom) is seen at end-stage PS70 in the mice given ICV injection of 25 µg ASO-20-37. H&E, acetate non-specific esterase, and NADH dehydrogenase staining. Scale bar: 200 µm.

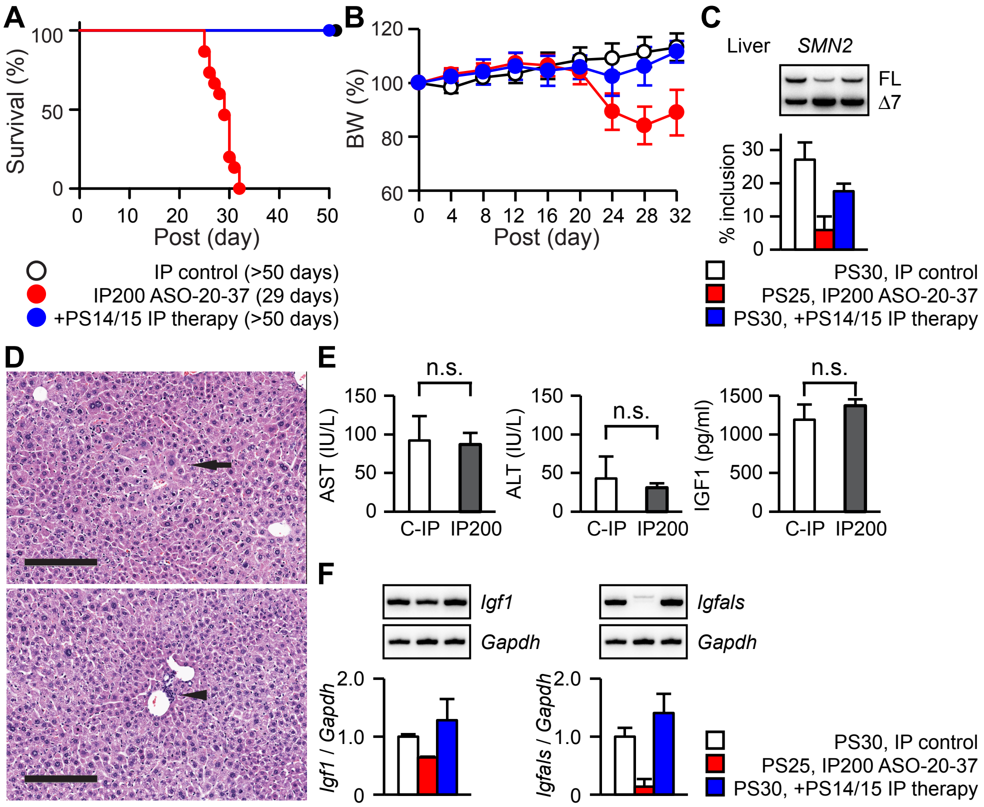

Figure S5. Amelioration by systemically administered therapeutic ASO. 200 mg/kg/d therapeutic ASO-10-27 was injected intraperitoneally 14 and 15 days after IP injection of 200 mg/kg/d ASO-20-37. (**A**) Extended survival by ASO-10-27. Control mice (n = 10), mice without therapy (n = 15), and with IP injection of ASO-10-27 (n = 10) were analyzed. (**B**) Retained body weight. Control mice (n = 10), mice without therapy (n = 12), and with IP injection of ASO-10-27 (n = 10) were analyzed. The median survival days after the first injection of ASO-20-37 are given in parentheses for each group. (**C**) Increased exon 7 inclusion in *SMN2* in P30 liver (n = 3). (**D**) Ameliorated histological change in liver at PS30. There was only mild hepatocellular necrosis with sporadic enlarged cells (arrow), and inflammatory cell infiltration (arrowhead), particularly neutrophils. H&E staining. Scale bar: 200 μm. (**E**) Retained serum AST and ALT levels (n = 5. n.s., P = 0.7732 and 0.4442, respectively) and IGF1 levels (n = 5. n.s., P = 0.1472). (**F**) Retained mRNA levels of hepatic *Igf1* and *Igfals* at PS30 (n = 3). For *Igfals* in the mice treated with ASO-20-37 only, the lower band in each doublet was quantitated. IP control or IP200 ASO-20-37: IP injection of 200 mg/kg/d control ASO or ASO-20-37 for 2 days, respectively; +PS14/15 IP therapy: IP injection of 200 mg/kg/d ASO-20-37 for 2 days and IP injections of 200 mg/kg/d ASO-10-27 at PS14 and PS15. We analyzed data using two-tailed *t*-tests, except for the logrank test for survival analysis.

Table S1. Sequences of the uniform 2'-MOE ASOs with phosphorothioate backbone and 5-methyl cytosines used in this study.

Antisense oligonucleotide	Sequence
Control ASO	5´-TCATTTGCTTCATACAGG-3´
ASO-20-37	5´-GTGAGCACCTTCCTTCTT-3´
ASO-10-27	5´-TCACTTTCATAATGCTGG-3´

Table S2. Echocardiographic measurements before and 25 days after IP injection of control ASO or ASO-20-37.

Female		IP control (n = 3)				IP (n = 5)			
		Pre-surgery		PS25		Pre-surgery		PS25	
	Units	Average	SD	Average	SD	Average	SD	Average	SD
HR	b/min	540	32	486	2	513	16	459	23
IVS; d	mm	0.12	0.02	0.15	0.02	0.12	0.02	0.18	0.03
LVID; d	mm	3.52	0.07	3.78	0.18	3.60	0.09	3.21	0.14
LVPW; d	mm	0.81	0.06	0.99	0.08	0.82	0.06	1.03	0.10
IVS; s	mm	0.11	0.02	0.16	0.03	0.11	0.02	0.21	0.03
LVID; s	mm	2.01	0.07	1.97	0.23	2.09	0.10	1.76	0.17
LVPW; s	mm	1.24	0.08	1.44	0.12	1.24	0.07	1.47	0.10
LV Vol; d	µl	51.67	2.37	61.83	6.71	54.17	3.44	42.29	4.62
LV Vol; s	µl	13.00	1.20	12.89	3.54	14.38	1.74	9.69	2.32
% EF	%	75.00	2.28	79.81	4.07	73.79	2.31	77.43	4.50
% FS	%	43.01	2.09	48.06	4.08	41.98	2.03	45.16	4.14
LV Mass	mg	47.65	4.13	68.92	5.93	49.51	4.61	55.64	6.34

Male		IP control (n = 3)				IP (n = 5)			
		Pre-surgery		PS25		Pre-surgery		PS25	
	Units	Average	SD	Average	SD	Average	SD	Average	SD
HR	b/min	548	43	474	10	541	29	452	32
IVS; d	mm	0.11	0.02	0.14	0.02	0.11	0.02	0.47	0.04
LVID; d	mm	3.70	0.09	3.75	0.14	3.63	0.07	2.85	0.14
LVPW; d	mm	0.86	0.07	0.92	0.05	0.84	0.05	1.08	0.09
IVS; s	mm	0.11	0.02	0.14	0.03	0.12	0.02	0.55	0.04
LVID; s	mm	2.11	0.08	2.17	0.16	2.14	0.08	1.56	0.11
LVPW; s	mm	1.32	0.05	1.41	0.06	1.28	0.05	1.48	0.07
LV Vol; d	µl	58.07	3.30	60.42	5.34	55.59	2.56	32.21	3.57
LV Vol; s	µl	14.75	1.65	15.81	2.79	15.18	1.28	7.27	1.26
% EF	%	74.84	2.17	74.09	3.23	73.96	2.13	78.22	2.89
% FS	%	43.06	1.84	42.39	2.82	41.49	1.87	45.72	2.78
LV Mass	mg	54.30	4.62	61.74	5.17	50.73	3.82	65.04	19.16

HR, heart rate; IVS, interventricular septum; LVID, left ventricular end-diastolic internal dimension; LVPW, left ventricular posterior wall; % EF, ejection fraction: % of (end-diastolic volume - end-systolic volume) / end-diastolic volume; % FS, fractional shortening: % of (end-diastolic dimension - end-systolic dimension) / end-diastolic dimension; d / s, systolic / diastolic; Vol, volume.

IP control, 2-day IP injection of 200 mg/kg/d control ASO; IP, 2-day IP injection of 200 mg/kg/d ASO-20-37.
